# Crown tissue proportions and enamel thickness distribution in the Middle Pleistocene hominin molars from Sima de los Huesos (SH) population (Atapuerca, Spain)

**DOI:** 10.1371/journal.pone.0233281

**Published:** 2020-06-08

**Authors:** Laura Martín-Francés, María Martinón-Torres, Marina Martínez de Pinillos, Cecilia García-Campos, Clément Zanolli, Priscilla Bayle, Mario Modesto-Mata, Juan Luis Arsuaga, José María Bermúdez de Castro

**Affiliations:** 1 CNRS, MCC, PACEA, UMR 5199, Univ. Bordeaux, Bordeaux, France; 2 Centro Nacional de Investigación sobre la Evolución Humana, Burgos, Spain; 3 Anthropology Department, University College London, London, United Kingdom; 4 Equipo Primeros Pobladores de Extremadura, Casa de la Cultura Rodríguez Moñino, Cáceres, Spain; 5 Centro Mixto Universidad Complutense de Madrid - Instituto de Salud Carlos III de Evolución y Comportamiento Humanos, Madrid, Spain; 6 Departamento de Paleontología, Facultad de Ciencias Geológicas, Universidad Complutense de Madrid, Madrid, Spain; Ecole Normale Supérieure de Lyon, FRANCE

## Abstract

Dental enamel thickness, topography, growth and development vary among hominins. In *Homo*, the thickness of dental enamel in most Pleistocene hominins display variations from thick to hyper-thick, while Neanderthals exhibit proportionally thinner enamel. The origin of the thin trait remains unclear. In this context, the Middle Pleistocene human dental assemblage from Atapuerca-Sima de los Huesos (SH) provides a unique opportunity to trace the evolution of enamel thickness in European hominins. In this study, we aim to test the hypothesis if the SH molar sample approximates the Neanderthal condition for enamel thickness and/or distribution. This study includes 626 molars, both original and comparative data. We analysed the molar inner structural organization of the original collections (n = 124), belonging to SH(n = 72) and modern humans from Spanish origin (n = 52). We compared the SH estimates to those of extinct and extant populations of the genus *Homo* from African, Asian and European origin (estimates extracted from literature n = 502). The comparative sample included maxillary and mandibular molars belonging to *H*. *erectus*, East and North African *Homo*, European Middle Pleistocene *Homo*, Neanderthals, and fossil and extant *H*. *sapiens*. We used high-resolution images to investigate the endostructural configuration of SH molars (tissue proportions, enamel thickness and distribution). The SH molars exhibit on average thick absolute and relative enamel in 2D and 3D estimates, both in the complete crown and the lateral enamel. This primitive condition is shared with the majority of extinct and extant hominin sample, except for Neanderthals and some isolated specimens. On the contrary, the SH molar enamel distribution maps reveal a distribution pattern similar to the Neanderthal signal (with thicker enamel on the lingual cusps and more peripherally distributed), compared to *H*. *antecessor* and modern humans. Due to the phylogenetic position of the SH population, the thick condition in molars could represent the persistence of the plesiomorphic condition in this group. Still, more data is needed on other Early and Middle Pleistocene populations to fully understand the evolutionary meaning of this trait.

## Introduction

The European Middle Pleistocene record is central to understand the timing and pattern of the emergence of the Neanderthals [1–3]. Unfortunately, the human assemblages for this period are mainly composed by isolated, chronologically and geographically scattered remains. Therefore, the Atapuerca-Sima de los Huesos (SH) population provides a unique opportunity to trace the Neanderthal signature in the Middle Pleistocene. The SH site is a small cavity of 8m^2^ x 4m^2^ belonging to the Cueva mayor-Cueva del Silo karst system. To date, the human fossil assemblage comprises more than 6500 remains from at least 28 individuals recovered from a single stratigraphic level, LU6, [1]. A suite of dating methods, including U-series, OSL and ESR, provided a minimum age of ~427 ka [1, 4, 5]. Classically assigned to *H*. *heidelbergensis* species [6–9], the assignment of SH population to this controversial taxon [2, 10, 11] is being reconsidered [1]. In particular, the possible exclusion of SH population is due to the lack of shared cranial features with other European Middle Pleistocene fossils, such as Ceprano (see below) and Arago [1], and the noticeable differences between the Mauer and the SH mandibular samples [12]. On the contrary, morphological and paleogenetic studies evince the affinities between the SH population and Neanderthals [1, 13–18]. Moreover, the increasing evidence shed by the study of the SH sample [1, 15, 16, 19] is also challenging the accretion model for the Neanderthals [3, 20]. The degree of expression of some Neanderthal features observed in the SH dentition [15, 17, 21, 22] makes difficult to interpret the evolution of the Neanderthal features in a chronological sequence. Finally, while the mitochondrial DNA revealed that SH is the closest group related to the eastern Siberian-Denisovans [23], the nuclear DNA [18] confirmed the long maintained assumption that SH belonged to the Neanderthal clade [1, 6, 7, 17].

In addition, other dental investigations suggest the existence of more than one hominin lineage during the Middle Pleistocene. Bermúdez de Castro and colleagues [16] showed that while SH teeth are practically identical to those from Neanderthals, the Middle Pleistocene sample from Arago exhibits a higher number of primitive traits. Similarly, the penecontemporaneous Italian sites of Fontana Ranuccio and Visogliano, ~450 ka, also revealed morphological and metrical similarities with SH and Neanderthals [24].

Still vigorous debate revolves around the phylogenetic interpretation of the European Middle Pleistocene groups and their relationship with Neanderthals. In this context, the Middle Pleistocene SH population represents a unique opportunity to contribute to this debate. In this study, we characterise the SH molar tissue proportions, and discuss its taxonomic and phylogenetic interpretation.

Enamel is often discussed in paleoanthropological literature, particularly with regard to differences in thickness, distribution and growth between Neanderthals and modern humans [25–32]. Until now, the taxonomic value of enamel thickness is limited to the Neanderthals [25, 29, 33]. In particular, the relative thin enamel condition documented in Neanderthals is likely linked to odontogenetic mechanisms, such as a faster developmental trajectory and a more complex topography and larger surface of the EDJ [34–37]. On the contrary, studies on modern humans relate their relative thick enamel to a unique odontogenetic process and to the extreme dental reduction [29, 38–40]. Previous results on crown tissue proportions in *H*. *antecessor* (from the TD6 level of the Gran Dolina site, Atapuerca) and SH dentition [41, 42] showed that TD6 molars shared with Neanderthals some histological aspects, such as the lateral enamel thickness and the enamel thickness distribution [41]. In addition, García-Campos and colleagues [42] described a Neanderthal-like, thin pattern of enamel thickness in TD6 and SH canines. Considering that Early and Middle Pleistocene populations of Atapuerca (TD6 and SH) already exhibit Neanderthal-like aspects of enamel thickness, here we test the hypothesis if the SH molar sample exhibits some Neanderthal aspects of enamel thickness. To do so, we estimated linear, 2D, and volumetric, 3D, enamel thickness on the SH molar sample and compare it to fossil and extant populations.

### Material

This study includes 626 molars, including original and comparative data (Table 1). We assessed the wear degree following Molnar’s categories [43]. We selected molars exhibiting wear scores between 1 (no wear) and 4 (dentine patches). Due to the lack of a reproducible protocol to reconstruct enamel cusps and dentine horns in 3D, we excluded molars exhibiting wear 3 and 4 from the 2D and 3D, total crown volume analyses, but we included them in our investigation of the lateral tissue proportions in which the occlusal enamel is removed.

**Table 1:** Fossil and recent comparative samples used for crown 2D and 3D complete crown and lateral 3D measurements.

Samples	N	Tooth	Specimens	References
*H*. *antecessor* (TD6)	4	M^1^	Atapuerca-Gran Dolina: ATD6-10, ATD6-11, ATD6-69, ATD6-103	[41]
*H*. *erectus* (HER)	2		Apothecary, China: CA770	[44]
			Sangiran: NG91-G10n°1	[45]
North African *Homo* (NAH)	1		Tighenif: UM1	[46]
Sima de los Huesos (SH)	8		Atapuerca-SH: AT-20, AT-26, AT-196, AT-812, AT-959, AT-2071, AT-3177, AT-5804	Original data
European Middle Pleistocene *Homo* (EMPH)	2		Steinheim	[29]
			Visogliano	[24]
Neanderthal (NEA)	11		Engis2	[25]
			El Sidrón: SR1105	
			Le Moustier1	
			Scladina: SCLA_4A_4	
			La Quina: H18	[41]
			Krapina: KRD134, KRD101, D136, D171, D174, D16	
Modern humans (MH)	57		Modern humans from South Africa, North America and Europe (n = 39)	[25, 29, 47, 48]
			Modern humans from Europe (n = 8)	[41]
			Modern humans from Spain (n = 10)	Original data
*H*. *antecessor* (TD6)	2	M^2^	Atapuerca-Gran Dolina: ATD6-12, ATD6-69	[41]
*H*. *erectus* (HER)	2		Apothecary Collection China: CA 771	[29]
			Hexian: PA833	[49]
North African *Homo* (NAH)	1		Thomas Quarry 3	[29]
Sima de los Huesos (SH)	12		Atapuerca-Sima de los Huesos: AT-12, AT-824, AT-817, AT-15, AT-170, AT-960, AT-822, AT-2175, AT-815, AT-588, AT-4336, AT-6215	Original data
European Middle Pleistocene *Homo* (EMPH)	2		Steinheim	[29]
			Visogliano3	[24]
Neanderthal (NEA)	14		El Sidrón: SR332, SR4, SR531, SR551	[25]
			Le Moustier1	
			Scladina: SCLA_4A_3	
			Spy: Spy I	[50]
			La Quina: H18	Original data from SFR
			Krapina: KRD98, D96, D135, D165, D166, D169	Original data from Nespos
Fossil *H*. *sapiens* (FHS)	1		Qafzeh 15	[29]
Modern humans (MH)	40		Modern humans from South Africa, North America and Europe (n = 27)	[25, 29, 47, 48]
			Modern humans from Europe (n = 5)	[41]
			Modern humans from Spain (n = 10)	Original data
*H*. *erectus* (HER)	3	M^3^	Sangiran4	[41]
			Sangiran: NG0802.1	[45]
			Zhoukoudian: PMU M3550	[51]
Sima de los Huesos (SH)	15		Atapuerca-SH: AT-10, AT-194, AT-601, AT-805, AT-826, AT-819, AT-3181, AT-1417, AT-2393, AT-3183, AT-5082, AT-5292, AT-274, AT-602, AT-6215	Original data
Neanderthal (NEA)	14		El Sidrón: SR407, SR741, SR621	[25]
			Le Moustier 1Scladina: SCLA-4A_3	
			Spy: I, II, III	[50]
			Las Palomas 51	[28]
			Krapina: D97, D109, D162, D163, D170	
				Original data from Nespos
Modern humans (MH)	80		Modern humans from South Africa, North America and Europe (n = 67)	[25, 29, 47, 48]
			Modern humans from Europe (n = 5)	[41]
			Modern humans from Spain (n = 8)	Original data
*H*. *antecessor* (TD6)	4	M_1_	Atapuerca-Gran Dolina: ATD6-5, ATD6-94, ATD6-112, ATD6-96	[41]
East African *Homo* (EAH)	1		Buia: MA 93	[52]
North African *Homo* (NAH)	1		Tighenif	[41]
*H*. *erectus* (HER)	1		Sangiran: NG92.2	[41]
Sima de los Huesos (SH)	13		Atapuerca-SH: AT-2, AT-3933, AT-101, AT-141, AT-272, AT-829, AT-1759, AT-2276, AT-2438, AT-4318, AT-21, AT-576, AT-561	Original data
European Middle Pleistocene *Homo* (EMPH)	2		Fontana Ranuccio: FR1R	[24]
			Montmaurin-La Niche	[53]
Neanderthal (NEA)	22		Roc de Marsal (2)	[25]
			Engis 2	
			Ehringsdorf: G-1048-69	
			El Sidrón: SR755, SR540	
			Le Moustier 1	
			Regoudou 1	
			Scladina: SCLA_4A_1	
			Abri Suard: S5, S49, S14-7	
			Abri Bourgeois-Delaunay: BDJ4C9	
			Combe Grenal: CG IV	
			Krapina: KRP53, KRP54, KRP55, KRPD80, D77, D79, D81, D105	[41]
Modern humans (MH)	81		Modern humans from South Africa, North America and Europe (n = 55)	[25, 29, 47, 48]
			Modern humans from Europe (n = 13)	
			Modern humans from Spain (n = 13)	[41]
				[54] and original data
*H*. *antecessor* (TD6)	3	M_2_	Atapuerca-Gran Dolina: ATD6-5, ATD6-144, ATD6-96	[41]
North African *Homo* (NAH)	1		Tighenif: Tighenif_2	[46]
*H*. *erectus* (HER)	4		Sangiran: NG0802.3, NG92.3, NG92D6ZE57s/d76, NG0802.2	[45]
Sima de los Huesos (SH)	12		Atapuerca-SH: AT-3179, AT-169, AT-271, AT-284, AT-1761, AT-941, AT-946, AT-2270, AT-2396, AT-3176a, AT-3176b, AT-6579	Original data
European Middle Pleistocene *Homo* (EMPH)	1		Montmaurin-La Niche	[53]
Neanderthal (NEA)	13		Abri Suard: S36	[25][41]
			Krapina: KRD6, KRD10 (2), KRP55	
			KRP54, D86, D107, D1	
			Le Moustier 1	
			Regourdou 1 (2)	
			Scladina: SCLA_4A_1	
Modern humans (MH)	89		Modern humans from South Africa, North America and Europe (n = 46)	[25, 29, 47, 48]
			Modern humans from Europe (n = 16)	[41, 55]
			Modern humans from Spain (n = 27)	[54] and original data
*H*. *antecessor* (TD6)	3	M_3_	Atapuerca-Gran Dolina: ATD6-5, ATD6-113, ATD6-96	Original data
North African *Homo* (NAH)	1		Tighenif: Tighenif_2	[46]
*H*. *erectus* (HER)	1		Sangiran: NG9107.2	[45]
Sima de los Huesos (SH)	12		Atapuerca-SH: AT-30, AT-811, AT-143, AT-1468, AT-599, AT-942, AT-1959, AT-2438, AT-2273, AT-2777, AT-3182, AT-3943	Original data
European Middle Pleistocene *Homo* (EMPH)	3		Mauer	[29]
			Mala Balanica: BH-1	[56]
			Montmaurin-La Niche	[53]
Neanderthal (NEA)	15		Abri Suard: S36, S43	[25, 41]
			Krapina: KRD9, KRP57, KRPD85, KRD5, KRD7, KDR106	
			Le Moustier 1 (2)	
			Regourdou 1 (2)	
			La Quina: Q760-H9	
			Abri Bourgeois-Delaunay: BD01	
			Combe Grenal: CG XII	
Modern humans (MH)	72		Modern humans from South Africa, North America and Europe (n = 44)	[25, 29, 47, 48]
			Modern humans from Europe (n = 8)	[41]
			Modern humans from Spain (n = 20)	[54] and original data

*Please note that in Smith et al.,[47] the modern humans’ data does not include individual values, therefore we only employed it for comparative purposes but it was not possible to include it in the boxplots or statistical analyses.

To date, the SH dental assemblage includes 81 molars. In this study, we analysed the tissue proportions of all but nine molars that exhibit either extensive damage to the crown or advanced occlusal wear (score 5 Molnar’s classification [43]). Therefore, we analysed 72 molars, 35 maxillary and 37 mandibular, belonging to SH population (Table 1). For comparative purposes, the study included data from 554 molars belonging to extinct and extant populations of the genus *Homo* of African, Asian and European origin. Although most of the data of the comparative material was extracted from the literature (Table 1), we also included original data such as the Neanderthal maxillary M^3^ from Krapina (available online on the NESPOS database, 2019) and modern humans from Spain.

## Methods

### Scanning of the samples

Microtomographic scanning (μCT) of the fossil and recent comparative samples was performed in two European facilities. The SH isolated molars were scanned with Scanco Medical Micro-CT80 system, using the following parameters: 70 kV and 114 mA, 0.1 Al filter and resulting isometric voxel size 18μm. Since 2015 with the acquisition of a new equipment at CENIEH, part of the modern samples (including both isolated molars and teeth included in the jaws), were scanned with a GE 103 Phoenix v/tome/x_s 240 instrument with a different set of parameters ranging from 120-140kV and 140μA, 0.2 Cu filter and resulting isometric voxel size ranging from 27 to 36μm. The other part of the modern human collection was scanned using a μCT located in the Multidisciplinary Laboratory of the International Centre for Theoretical Physics (ICTP) in Trieste, Italy [57]. All scans were performed with two 0.1 mm copper filters at a voltage of 100–120 kV and amperage of 110–140 μA, resulting in a voxel size ranging from 17 to 21μm.

### Virtual segmentation

Using Amira v.6.3.0 (FEI Inc.) and ImageJ v.1.46 [58], a semi-automatic threshold-based segmentation was carried out following the half-maximum height method (HMH; [59]) and the region of interest thresholding protocol (ROI-Tb; [60], taking repeated measurements on different slices of the virtual stack ([61]).

The virtual molar sectioning was performed following the protocol described in Olejniczak and colleagues [25], instead of that developed by Smith et al., [30], and based on a 2D plane perpendicular to the developmental axis of the crown. Comparisons between the methods did not reveal significant differences in average or relative enamel thickness [30]. That is, we imported the μCT image stack into Amira (6.3.0, FEI Inc.) and rotated into anatomical position. Then, the tip of three dentine horns (protocone, paracone and metacone in the maxillary molars and protoconid, metaconid and hypoconid in the mandibular molars) were identified and the image stack was adjusted to intersect these three points of interest. Finally, a new plane perpendicular to the plane containing the three dentine horns was rotated to pass through the mesial dentine horns (protocone and paracone in the maxillary molars and protoconid and metaconid in the mandibular molars) ([25] and also see S1 Fig) 39 out of the 72 SH molars were discarded for exhibiting wear score higher than two in Molnar’s classification [43]. Therefore, we assessed enamel thickness from virtual 2D mesial cross-section planes in 48 SH molars (25 maxillary and 23 mandibular; see Table 2 for the number of specimens in each molar type) using Amira (6.2, FEI Inc.) and ImageJ (1.51, NIH). In each mesial plane, we measured the enamel (c) and dentine cap (b, including the pulp) areas (in mm^2^), adding up into the total crown area (a, in mm^2^), and the enamel-dentine junction (EDJ) length (d, in mm). We calculated the average enamel thickness (AET = c/d), the relative enamel thickness (RET = 100*AET/(b^1/2^); [48, 62]) and the percentage of dentine and pulp in the molar crown (b/a = 100*b/a in %). Inter- and intra-observer error was assessed by two of the authors. They performed the complete process, including orientation of the specimen, mesial plane definition, measures of the variables in each SH specimen. Each set of measurements was repeated in three alternative days, inter- and intra-observer error resulted in < 4%.

**Table 2:** 2D enamel thickness variables assessed in SH maxillary and mandibular molars and compared with extinct and extant specimens/populations (SH data in bold). Mean and range are given for the comparative sample when having more than two specimens. Individual values are given for the rest of comparative sample.

Sample	N	Tooth class		2D AET (mm)	2D RET	b/a*100
TD6	4	M^1^	Mean	1.12	17.14	64.98
			SD	0.06	1.15	2.4
			Range	1.07–1.21	16.07–18.38	62.16–67.72
**SH**	**3**		**Mean**	**1.04**	**16.76**	**63.82**
			**SD**	**0.02**	**0.64**	**1.36**
			**Range**	**1.03–1.06**	**16.03–17.21**	**62.81–65.37**
HER	2		Mean	1.31	18.67	62.22
			SD	0.02	0.56	0.7
			Range	1.30–1.32	18.28–19.07	61.72–62.72
MPEH_St	1			1.08	16.9	63.81
NEA	5		Mean	1.03	15.50	65.68
			SD	0.10	1.22	1.88
			Range	0.93–1.19	13.80–16.93	63.88–68.04
*MH	37		Mean	1.22	18.75	62.85
			SD	0.12	2.08	2.67
			Range	0.98–1.50	13.95–23.86	57.16–68.98
MH	12		Mean	1.07	18.13	63.11
			SD	0.18	2.66	3.06
			Range	0.84–1.45	14.46–22.71	58.31–67.58
		M^2^				
TD6	2		Mean	1.39	21.74	60.65
			SD	0.18	2.09	1.83
			Range	1.27–1.52	20.26–23.22	59.35–61.94
**SH**	**8**		**Mean**	**1.18**	**19.62**	**60.55**
			**SD**	**0.09**	**1.69**	**2.65**
			**Range**	**1.06–1.28**	**16.88–22.22**	**56.36–64.91**
HER	2		Mean	1.49	21.47	
			SD	0.02	2.9	
			Range	1.48–1.51	19.42–23.52	
NEA	6		Mean	1.2	18.12	62.96
			SD	0.08	1.84	2.75
			Range	1.13–1.29	15.65–20.85	59.17–66.90
MPEH_St	1			1.2	17.13	65.51
MPAH_TQ	1			1.42	18.81	63.94
FHS_Qz	1			1.31	19.84	62.69
*MH	25		Mean	1.4	21.59	60.05
			SD	0.17	3.13	3.46
			Range	1.13–1.76	16.49–28.03	53.80–66.45
MH	12		Mean	1.32	21.75	59.59
			SD	0.27	4.83	5.71
			Range	0.82–1.80	13.72–29.59	49.69–70.02
		M^3^				
MPEH_St	1			1.25	21.59	60.23
MPAH_TQ	1			1.56	22.96	59.49
**SH**	**14**		**Mean**	**1.3**	**23.84**	**54.49**
			**SD**	**0.13**	**2.07**	**3.64**
			**Range**	**1.04–1.46**	**19.88–27.56**	**47.48–59.92**
NEA	6		Mean	1.1	16.45	66.4
			SD	0.19	1.94	2.88
			Range	0.79–1.35	13.82–18.49	63.51–70.08
*MH			Mean	1.38	21.75	59.93
			SD	0.14	2.85	3.23
			Range	1.18–1.95	17.02–30.01	52.35–66.13
MH	11		Mean	1.30	22.38	59.17
			SD	0.20	4.10	5.57
			Range	1.01–1.64	16.63–28.99	52.58–68.54
		M_1_				
TD6	4		Mean	1.17	19.92	61.25
			SD	0.16	3.26	5.03
			Range	1.00–1.38	16.90–23.12	55.44–65.89
EAH	1			1	16.17	67.25
**SH**	**1**			**1.00**	**17.96**	**60.51**
NEA	13		Mean	1.00	15.88	65.62
			SD	0.07	1.69	2.59
			Range	0.92–1.18	13.77–20.46	60.49–69.28
*MH	55		Mean	1.07	16.99	64.48
			SD	0.13	2.29	3.073
			Range	0.80–1.40	11.76–22.62	59.19–72.65
MH	11			1.11	19.47	61.09
				0.09	1.93	2.24
				0.96–1.26	16.85–22.35	58.11–64.99
TD6	4	M_2_	Mean	1.16	21.60	60.01
			SD	0.17	4.06	5.56
			Range	1.02–1.37	17.65–27.25	52.53–65.98
HER	4		Mean	1.27	22.15	59.03
			SD	0.09	2.71	3.10
			Range	1.19–1.38	18.56–24.93	56.04–63.15
NAH_Tf	1			1.19	17.29	65.43
MPEH_M-LN	1			1.16	18.72	63.16
**SH**	**10**		**Mean**	**1.20**	**22.51**	**59.79**
			**SD**	**0.11**	**2.58**	**2.31**
			**Range**	**1.02–1.41**	**19.57–28.30**	**54.95–62.40**
NEA	9		Mean	1.01	15.59	67.25
			SD	0.07	0.91	1.28
			Range	0.90–1.19	14.21–22.24	65.28–69.69
*MH	45		Mean	1.19	20.51	60.78
			SD	0.14	2.93	3.249
			Range	0.94–1.55	14.85–27.66	53.21–67.80
MH	21		Mean	1.25	22.14	58.73
			SD	0.15	3.11	3.56
			Range	1.02–1.53	16.22–28.58	50.76–66.39
TD6	3	M_3_	Mean	1.14	24.00	58.11
			SD	0.05	4.50	5.02
			Range	1.09–1.20	21.16–29.19	52.34–61.47
HER	1			0.97	19.87	60.25
**SH**	**12**		**Mean**	**1.32**	**26.88**	**54.70**
			**SD**	**0.16**	**4.44**	**5.27**
			**Range**	**0.93–1.48**	**16.68–31.98**	**46.60–67.28**
MPEH_MR	1			1.27	21.6	
MPEH_M-LN	1			1.16	20.29	62.05
MPEH_BH	1			0.94	17.86	64.36
NEA	11		Mean	1.03	17.43	64.73
			SD	0.08	1.92	2.96
			Range			
*MH	44		Mean	1.24	21.63	59.31
			SD	0.15	2.99	3.15
			Range	0.98–1.67	17.22–31.84	50.82–64.61
MH	17		Mean	1.22	22.02	58.42
			SD	0.12	2.68	2.87
			Range	1.03–1.50	17.56–29.09	52.05–63.83

Upper molars. TD6: *H*. *antecessor* from Gran Dolina [41]. SH: Sima de los Huesos (original data). HER: *H*. *erectus* [29, 44, 45, 49]. EMPH: European Middle Pleistocene *Homo* (St: Steinheim [29]). MPAH: Middle Pleistocene African *Homo* (TQ: Thomas Quarry [29]). NEA: Neanderthals [25, 28]. FHS: fossil *H*. *sapiens* (Qz: Qafzeh [29]). *MH: modern humans [47]. *Please note that in Smith et al., [47] the modern humans’ data does not include individual values, therefore we only employed it for comparative purposes but it was not possible to include it in the boxplots or statistical analyses. MH: modern humans (original data).

Lower molars. TD6: *H*. *antecessor* from Gran Dolina [41]. EAH: East African *Homo* [52]. NAH: North African *Homo* (Tf: Tighenif [46]). HER: *H*. *erectus* [45]. SH: Sima de los Huesos (original data). EMPH: European Middle Pleistocene *Homo* (MR: Mauer [29]; M-LN: Mountmaurin [53]; BH: Mala Balanica [56]). NEA: Neanderthals [25, 28]. *MH: modern humans [47]. *Please note that in Smith et al., [47] the modern humans’ data does not include individual values, therefore we only employed it for comparative purposes but it was not possible to include it in the boxplots or statistical analyses. MH: modern humans [54] and original data.

Similarly, due to wear degree, we assessed 3D tissue proportions in 48 (out of the 72) SH molars (35 maxillary and 37 mandibular; see Table 3 for the number of specimens in each molar type). Using Amira (6.3.0, FEI Inc.) we performed the segmentation of the dental tissues (enamel, dentine and pulp). We used the semiautomatic tool, threshold-based segmentation, and manual corrections. We employed Olejniczak et al., [25] protocol for the definition of the cervical plane. That is, the plane is halfway between the most apical continuous ring of enamel and the plane containing the last hint of enamel. The following variables were measured and/or calculated: volume of the enamel (Ve in mm^3^); volume of the coronal dentine including the pulp enclosed in the crown (Vcdp in mm^3^); total volume of the crown, including the enamel, dentine and pulp (Vc in mm^3^); surface of the EDJ (SEDJ in mm^2^); percentage of dentine and pulp in the total crown volume (Vcdp/Vc = 100*Vcdp/Vc in %); 3D average enamel thickness (3D AET = Ve/SEDJ in mm) and, 3D relative enamel thickness (3D RET = 100*3D AET/(Vcdp^1/3^) a scale-free measurement) [25, 63].

**Table 3:** Mann-Whitney comparative statistical test (significant *p* values (0.05) in bold) for the 2D measurements in SH, Neanderthals and modern humans.

Groups	Tooth class	2D AET	2D RET	b/a
SH vs NEA	M^2^	0.56	0.12	0.15
SH vs MH		0.09	0.21	0.70
NEA vs MH		0.21	0.06	0.18
	M^3^	AET	RET	Vcdp/Vc
SH vs NEA		**0.02**	**0.00**	**0.00**
SH vs MH		0.74	0.31	0.08
NEA vs MH		0.07	**0.01**	**0.02**
	M_2_			
SH vs TD6		0.67	0.39	0.67
SH vs HER		0.25	1	0.57
SH vs NEA		**0.00**	**0.00**	**0.00**
SH vs MH		0.58	0.73	0.34
NEA vs MH		**0.00**	**0.00**	**0.00**
	M_3_			
SH vs NEA		**0.00**	**0.00**	**0.00**
SH vs MH		**0.03**	**0.00**	**0.01**
NEA vs MH		**0.00**	**0.00**	**0.00**

Upper molars: SH: Atapuerca-Sima de los Huesos (original data); NEA: Neanderthals from various sites [25, 28]; MH: modern humans [48 and original data]. Lower molars: TD6: *H*. *antecessor* from Atapuerca-Gran Dolina [41]; SH: Atapuerca-Sima de los Huesos (original data); NEA: Neanderthals from various sites [25, 28]; MH: modern humans [48 and original data].

In order to extract the largest amount of information of the SH molar collection, including the occlusal worn molars, we assessed lateral (non-occlusal) enamel thickness in the 72 molars of the SH population (25 maxillary and 23 mandibular; see Table 4 for the number of specimens in each molar type). In Amira (6.3.0, FEI Inc.) we defined the occlusal basin plane, a plane parallel to the cervical plane and tangent to the lowest enamel point of the occlusal basin. Following all material above the occlusal basin plane were removed and only the enamel, dentine and pulp between these two planes were measured [64, 65]. The following variables were measured and/or calculated: lateral volume of the enamel (LVe in mm^3^); lateral volume of the coronal dentine including the pulp enclosed in the crown (LVcdp in mm^3^); total lateral volume of the crown, including the lateral enamel, dentine and pulp (LVc in mm^3^); lateral surface of the EDJ (LSEDJ in mm^2^); percentage of dentine and pulp in the lateral crown volume (LVcdp/LVc = 100*LVcdp/LVc in %); 3D average enamel thickness (3D LAET = LVe/LSEDJ in mm) and, 3D lateral relative enamel thickness (3D LRET = 100*3D LAET/(LVcdp^1/3^) a scale-free measurement [24, 41].

**Table 4:** 3D enamel thickness, complete crown, variables assessed in SH maxillary and mandibular molars and compared with extinct and extant specimens/populations (SH data in bold). Mean and range are given for the comparative sample when having more than two specimens. Individual values are given for the rest of comparative sample.

Sample	N	Tooth class		3D AET	3D RET	Vcdp/Vc
TD6	2	M^1^	Mean	1.30	18.69	53.11
			SD	0.09	2.75	3.23
			Range	1.24–1.36	16.75–20.64	50.83–55.40
HER	1			1.13	16.30	54.67
SH	3		**Mean**	**1.30**	**19.65**	**51.87**
			**SD**	**0.01**	**1.44**	**1.78**
			**Range**	**1.28–1.30**	**18.00–20.65**	**50.63–53.91**
NEA	4		Mean	1.14	16.39	55.36
			SD	0.07	2.20	3.39
			Range	1.07–1.20	13.93–18.41	51.25–58.77
MH	13		Mean	1.14	18.61	54.54
			SD	0.20	3.67	4.68
			Range	0.84–1.58	12.63–23.52	47.57–61.52
		M^2^				
TD6	2		Mean	1.44	21.30	50.92
			SD	0.17	3.24	2.69
			Range	1.32–1.54	19.01–23.59	48.24–54.14
**SH**	**8**		**Mean**	**1.24**	**20.15**	**51.51**
			**SD**	**0.10**	**1.47**	**1.64**
			**Range**	**1.05–1.43**	**18.43–23.74**	**48.24–54.14**
NEA	6		Mean	1.10	15.91	58.18
			SD	0.13	2.94	4.48
			Range	0.97–1.33	13.24–20.88	50.70–62.80
MH	14		Mean	1.33	21.93	51.03
			SD	0.19	3.94	3.49
			Range	0.96–1.78	15.06–31.63	43.24–58.46
		M^3^				
HER	1			1.45	27.64	42.45
**SH**	**14**		**Mean**	**1.36**	**25.39**	**46.10**
			**SD**	**0.11**	**2.06**	**2.08**
			**Range**	**1.18–1.50**	**21.24–30.02**	**41.43–50.82**
NEA	9		Mean	1.03	15.62	58.47
			SD	0.14	2.05	3.54
			Range	0.75–1.18	11.61–18.43	54.06–66.11
MH	22		Mean	1.44	26.10	46.44
			SD	0.24	5.14	4.35
			Range	0.91–1.94	14.54–34.10	37.83–52.98
		M_1_				
TD6	3		Mean	1.13	16.16	56.89
			SD	0.13	1.42	2.80
			Range	0.98–1.23	14.97–17.74	53.90–59.45
NAH_Tf	1			0.93	12.01	62.78
EAH_MA93	1			1.02	14.74	55.96
**SH**	**1**			**1.11**	**17.74**	**54.23**
NEA	12		Mean	1.13	16.29	58.47
			SD	0.23	3.72	3.53
			Range	0.82–1.63	11.79–24.02	52.01–63.52
LV	1			1.34	20.14	51.95
MH	19		Mean	1.14	17.97	52.64
			SD	0.13	2.65	3.10
			Range	0.92–1.36	14.46–22.21	46.37–57.35
		M_2_				
TD6	4		Mean	1.27	19.85	51.70
			SD	0.09	4.26	6.60
			Range	1.16–1.37	16.36–26.04	42.14–56.80
HER	4		Mean	1.34	21.42	48.40
			SD	0.07	1.94	2.24
			Range	1.26–1.42	18.98–23.60	45.77–51.07
NAH	1			1.19	15.01	57.39
**SH**	**10**		**Mean**	**1.30**	**22.20**	**49.67**
			**SD**	**0.10**	**2.42**	**2.62**
			**Range**	**1.11–1.50**	**18.74–27.25**	**45.02–54.40**
MPEH_M-LN	1			1.36	21.58	50.63
NEA	11		Mean	1.06	15.17	59.50
			SD	0.16	2.69	4.63
			Range	0.81–1.32	11.88–20.92	50.97–67.53
MH	35		Mean	1.29	20.55	50.51
			SD	0.29	5.13	4.45
			Range	0.65–2.30	12.56–40.71	36.56–57.32
		M_3_				
TD6	3		Mean	1.29	25.74	44.63
			SD	0.08	8.62	10.02
			Range	1.19–1.35	18.66–35.33	33.67–53.33
HER	1			1.05	18.01	52.80
NAH_Tf	2		Mean	1.11	15.59	57.34
			SD	0.49	6.62	10.45
			Range	0.77–1.46	10.90–20.27	49.95–64.73
**SH**	**12**		**Mean**	**1.40**	**24.54**	**47.95**
			**SD**	**0.14**	**2.85**	**3.35**
			**Range**	**1.05–1.53**	**17.12–27.90**	**44.81–57.50**
MPEH_M-LN	1			1.46	23.23	48.32
NEA	11		Mean	1.14	17.31	57.77
			SD	0.19	3.30	6.08
			Range	0.82–1.41	12.74–22.28	49.11–68.63
MH	20		Mean	1.36	22.82	48.53
			SD	0.18	3.53	3.87
			Range	1.08–1.85	17.78–30.20	42.18–55.44

Upper molars: TD6: *H*. *antecessor* from Gran Dolina [41]. HER: *H*. *erectus* [45]. SH: Sima de los Huesos (original data). NEA: Neanderthals from various sites [25, 28, 50]. MH: modern humans [25, 41 and original data]. Lower molars: TD6: *H*. *antecessor* from Gran Dolina [41]. NAH: North African *Homo* (Tf: Tighenif [46]). EAH: East African *Homo* (MA93: Buia [52]). SH: Sima de los Huesos (original data). HER: *H*. *erectus* [45]. NEA: Neanderthals [25, 28]. LV: Lagar Velho (original data from Nespos). MH: modern humans [25, 41, 55 and original data].

### Statistical analyses

Since the number of SH specimens varies depending on the type of measurement (2D or 3D, complete or lateral crown), we employed a different statistical approach depending on the analysis. We employed the Adjusted Z-score test for the 2D and 3D comparative analysis of the SH upper and lower M1 due to the small SH sample size, three and one specimens respectively. Adjusted Z-scores [62, 63] of the three variables accounting for tissue proportions (AET, RET and percentage of dentine) were computed to compare 2D and 3D dental tissue proportions and enamel thickness values of the SH specimens to the means and standard deviations of the Neanderthal and two MH groups. The Adjusted Z-score test allows the comparison of unbalanced and reduced samples by using Student’s inverse t distribution. In these Z-scores the -1.0 to +1.0 interval comprises the 95% of the variation in the reference sample.

Additional statistical tests were performed with SPSS software (v. 20, IBM Corp.). The normal distribution was assessed using the Shapiro–Wilk Test. Since normality was not assumed in any of our samples, we used the non-parametric Mann–Whitney U-test for comparisons between groups represented by four or more specimens (as in Smith et al., [29]). Means were determined to be significantly different at the 0.05 level.

In addition, standard box and whisker plots were computed to represent three set of variables of linear and volume measurements (including 2D and 3D (complete cap and lateral) AET, RET and percentage of dentine) of the complete sample.

Additionally, and in order to visualize the enamel thickness topographic distribution in SH molar crowns we generated the chromatic maps using the surface distance module (SDM) in Amira (6.3.0, FEI Inc.). We specifically generated the chromatic maps in molars with a wear score of 2 or lower following Molnar’s classification [43] The SDM module computes several different distance measures between two triangulated surfaces. For each vertex of one surface it computes the closest point on the other surface (Amira 6.3.0, FEI Inc.). We generated the OES and EDJ surfaces independently and connected the OES and EDJ surfaces through the SDM module. The computed distances between the OES and the ESJ are defined by a chromatic scale from thinnest (blue) to thickest (red) [28, 66]. For comparative purposes, we also generated the chromatic maps of a selected sample of specimens of European and African origin, including: Neanderthal from Abri Suard (S14-7 M_1_ and S43 M_3_) and Krapina (D10-M_2_, D96-M^2^ and M^3^); La Quina-H18 (M^1^) modern humans (all molar classes); Eritrea (M_1_) and Tighenif (lower M_2_ and M_3_).

### Ethics statement

This study concerns the analysis of an original fossil human dental sample constituted by seventeen isolated specimens recovered from Atapuerca-Sima de los Huesos. The specimens are currently stored at the Centro Mixto de Evolución y Comportamiento Humanos (UCM-ISCIII), Madrid, Spain. Prof. Juan Luis Arsuaga, co-director of the Atapuerca Research Team, has made possible this study within the framework of a long-term scientific project in the field of paleoanthropology. The modern human collection comprises a Spanish sample deposited at the Escuela de Medicina Legal y Forense, Universidad Complutense de Madrid, Spain. Prof. Bernardo Perea gave access to this collection. All necessary permits were obtained for the described study, which complied with all relevant regulations, and accesses to these collections are granted through scientific collaborations. The data that support the findings of this study are available upon request from Profs. Arsuaga (UCM-ISCIII), Perea (UCM) and Martinón-Torres (CENIEH).

## Results

### Molar tissue proportions and enamel thickness

Tissue proportions assessed for the SH maxillary and mandibular molars and the comparative samples are shown in Tables 2–4 (see S1–S3 Tables for the complete set of variables and individual values on SH sample).

### 2D enamel thickness

**Fig 1:**
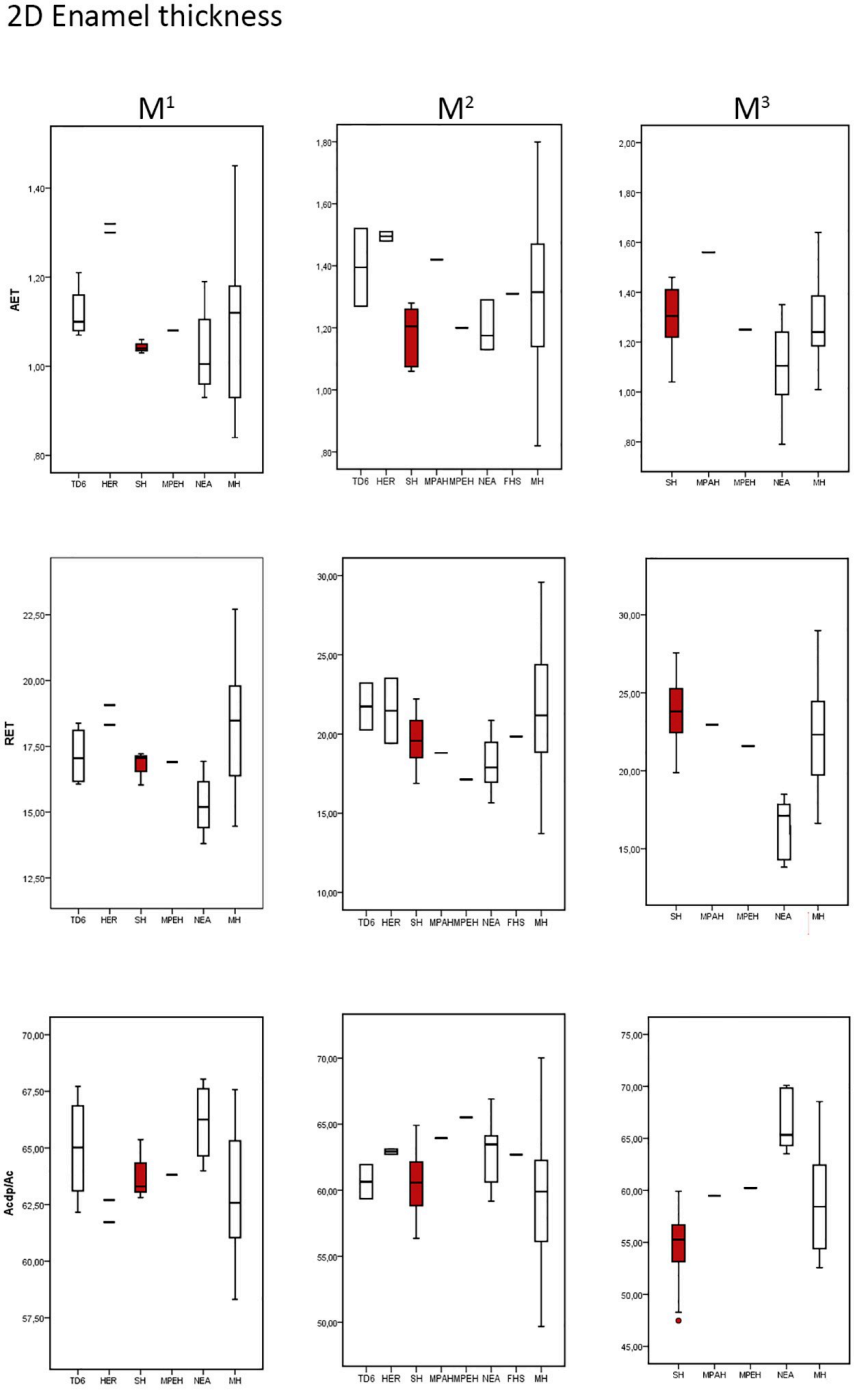
Box plots depicting 2D values. Values of the average enamel thickness (AET), relative enamel thickness (RET) and percentage of dentine and pulp (b/a) in the maxillary molars of the SH and the comparative specimens/samples.

**Fig 2:**
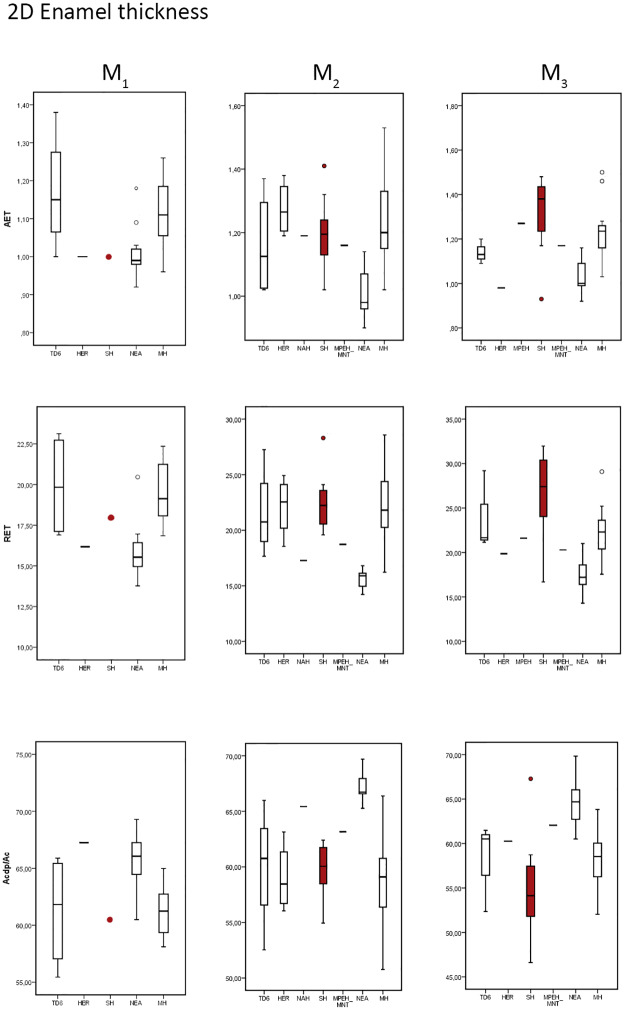
Box plots depicting 2D values. Values of the average enamel thickness (AET), relative enamel thickness (RET) and percentage of dentine and pulp (b/a) in the maxillary molars of the SH and the comparative specimens/samples.

The SH maxillary and mandibular molars follow an increasing pattern of enamel thickness from the M1 to the M3 (Table 2). Overall, the SH maxillary molars exhibit thick values of absolute and relative enamel thickness (2D AET and 2D RET) related to the low percentage of dentine (b/a*100) in the cap complex (Table 2 and S1 Table for SH individual values, Figs 1 and 2).

The SH maxillary molars, in terms of 2D AET, approximates the condition of European Middle Pleistocene specimen from Steinheim [29], and overlaps with *H*. *antecessor* population [41], Neanderthals [29] and modern humans [47, 48 and this study]. Regarding the TD6 population, the SH range encompasses its variability even exceeding the lower part of the TD6 variation range. The opposite is observed when comparing to Neanderthals, with SH population slightly exceeding the upper limits of the Neanderthal variation (Fig 1). When looking at 2D RET, the SH maxillary molars mean values approximate more the Steinheim value [29] for the upper M1, the fossil *H*. *sapiens* from Qafzeh15 value [29] for the upper M2 and the African Middle Pleistocene specimen from Thomas Quarry3 [29] for the upper M3. Still, the maxillary SH sample range overlaps with TD6, Neanderthal, and modern human populations, except for the Neanderthals upper M3 that shows lower values (Fig 1). For the 2D AET and 2D RET, the *H*. *erectus* sample, including the upper M1 from China (specimen CA770, [44]) and Sangiran (specimen NG91-G10n°1, [45]) and the upper M2 from Hexian (specimen PA833, [49]), is outside the range of variation of the SH population (Table 2 and Fig 1).

The results obtained for SH mandibular molars are similar to those described for the maxillary molars. However, the differences between the SH and Neanderthals and between SH and modern humans are larger for the M_3_ compared to the M^3^ (Table 2 and Fig 2). Broadly the SH mandibular molars estimates (Table 2) for 2D AET and 2D RET approximates the condition observed in the European Early Pleistocene population from TD6 [41] and modern human populations [47, 48 and original data]. The SH range overlaps and in some cases exceeds the range of these two populations (Fig 2). Regarding *H*. *erectus*, the SH mean sample of 2D RET is close to the Sangiran mean values reported by Zanolli [45].

**Fig 3:**
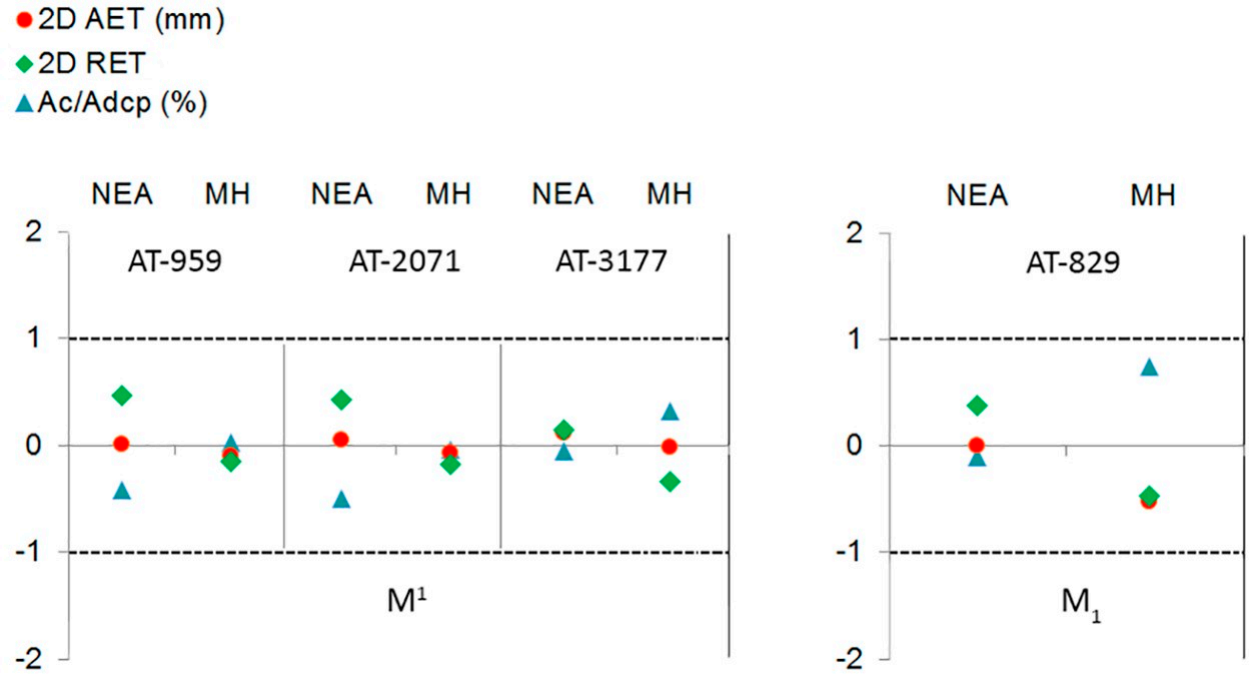
Adjusted Z score of the 2D variables (AET, RET and percentage of dentine). Assessed in the maxillary (specimens AT-959, AT-2071, AT-3177) and mandibular (specimen AT-829) molars from SH and compared to the variation expressed by Neanderthals (blue triangles) and modern humans (red circles). The solid line passing through zero represents the mean, and the other two lines correspond to the estimated 95% limit of variation expressed for the two comparative samples.

Fig 3 displays the results of the Adjusted Z-score test performed for the three upper (AT-959, AT-2071, and AT-3177) and one lower M1 (AT-829) from SH in comparison to the NEA and modern human populations for the three variables accounting for enamel thickness.

Overall, the three SH M^1^s fall within the 95% of the variation of the NEA and MH groups for all the variables (AET, RET and Acdp/Ac). Two (AT-959 and AT-2071) of the three SH specimens show closer relationship with the modern human population for the three variables. On the contrary, AT-3177 closely resembles the NEA condition for the RET and Acdp/Ac, while the SH specimen is closer to the modern population [48] and the Spanish collection (this study) for the AET variable. The SH M_1_ falls within the 95% of the Neanderthals and the modern human populations. Although SH specimen AT-829 closely resembles the Neanderthal condition for the three variables.

For the maxillary and mandibular M2 and M3 estimates, we first performed the non-parametric test Kruskal-Wallis to observe differences between three or more groups. Following, comparison between two groups was conducted using Mann-Whitney test (Table 3). We observed statistical differences between SH and Neanderthals at all molar positions (M^3^, M_2_ and M_3_), except for the M^2^, for the three variables accounting for enamel thickness. Differences between SH and MH were found solely for the M_3_ for all variables.

### 3D crown enamel thickness

As we observed for the 2D analysis, in SH sample the 3D enamel thickness follows an increasing trend from the M1 to M3. Similarly, the SH sample reflects the same thick pattern described for the 2D results (Table 4 and S2 Table for SH individual values), as the result of the low percentage of dentine (Vcdp/Vc) in the SH crown.

**Fig 4:**
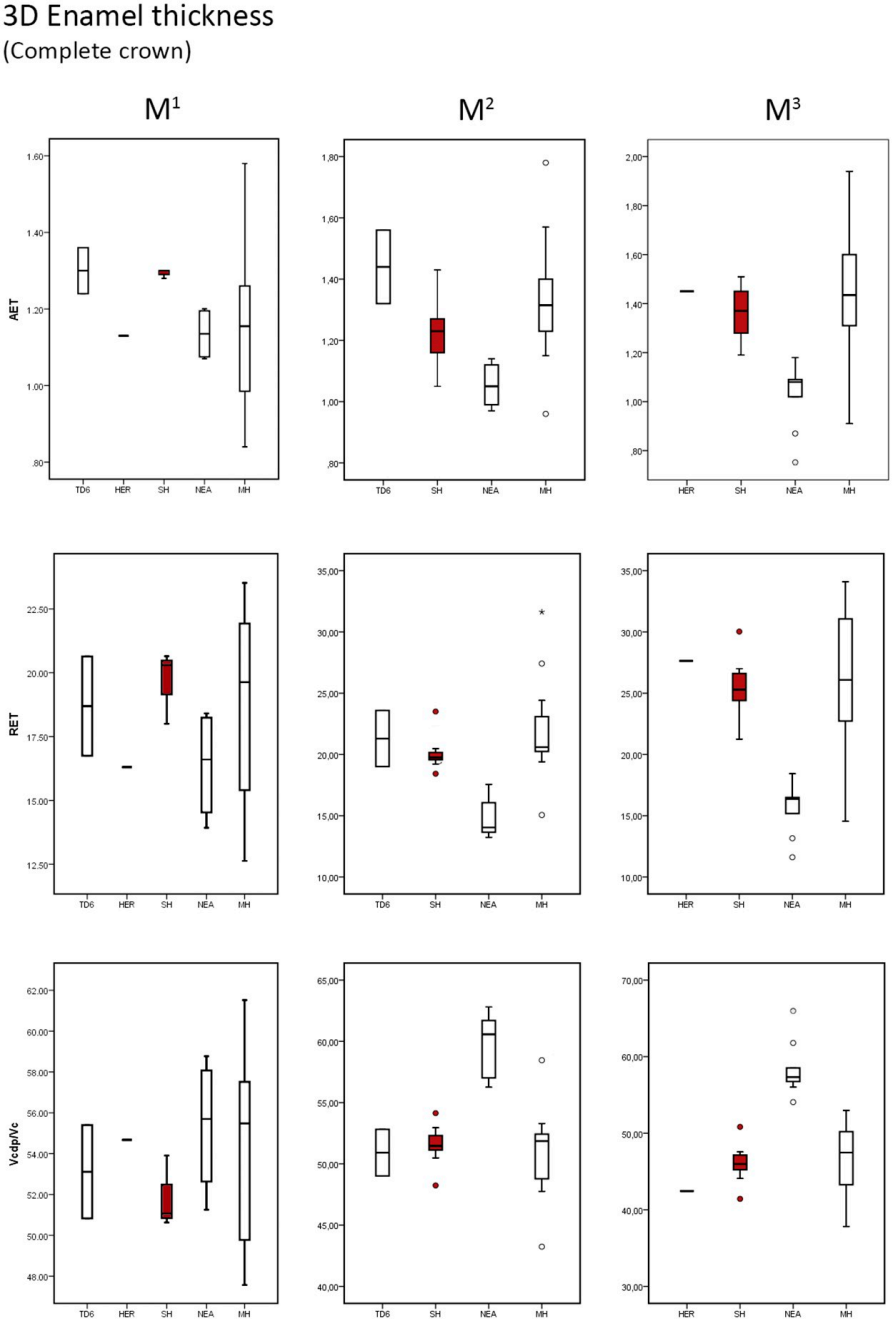
Box plots of the 3D, complete crown, values. 3D values depicting the average enamel thickness (3D AET), relative enamel thickness (3D RET) and the percentage of dentine and pulp in the total crown volume (Vcdp/Vc), in the maxillary molars of the SH and the comparative specimens/samples.

The SH maxillary estimates (Table 4) for the components of enamel thickness (3D AET, 3D RET and Vcdp/Vc) approximate the condition described for the European Early Pleistocene population of TD6 [41] and modern humans [25, 41 and original data] and differs from that of Neanderthals [25, 28, 50]. The SH variation range encompasses and exceeds in the lower part the range of variation seen in both TD6 and modern humans (Fig 4). Intergroup differences between Neanderthals and SH are observed at some metameric positions. More precisely, the SH range of variation for the 3D RET and Vcdp/Vc overlaps on the upper M1, while for the upper M2 and M3 there is no overlap between the SH and Neanderthal populations (Fig 4). Although the *H*. *erectus* specimen from Sangiran (NG0802.1, [45]) results for all variables are higher than the mean values of SH (this study) and modern humans [25, 41 and original data], still the Sangiran estimates are within the range of variation of both populations (Table 4 and Fig 4).

**Fig 5:**
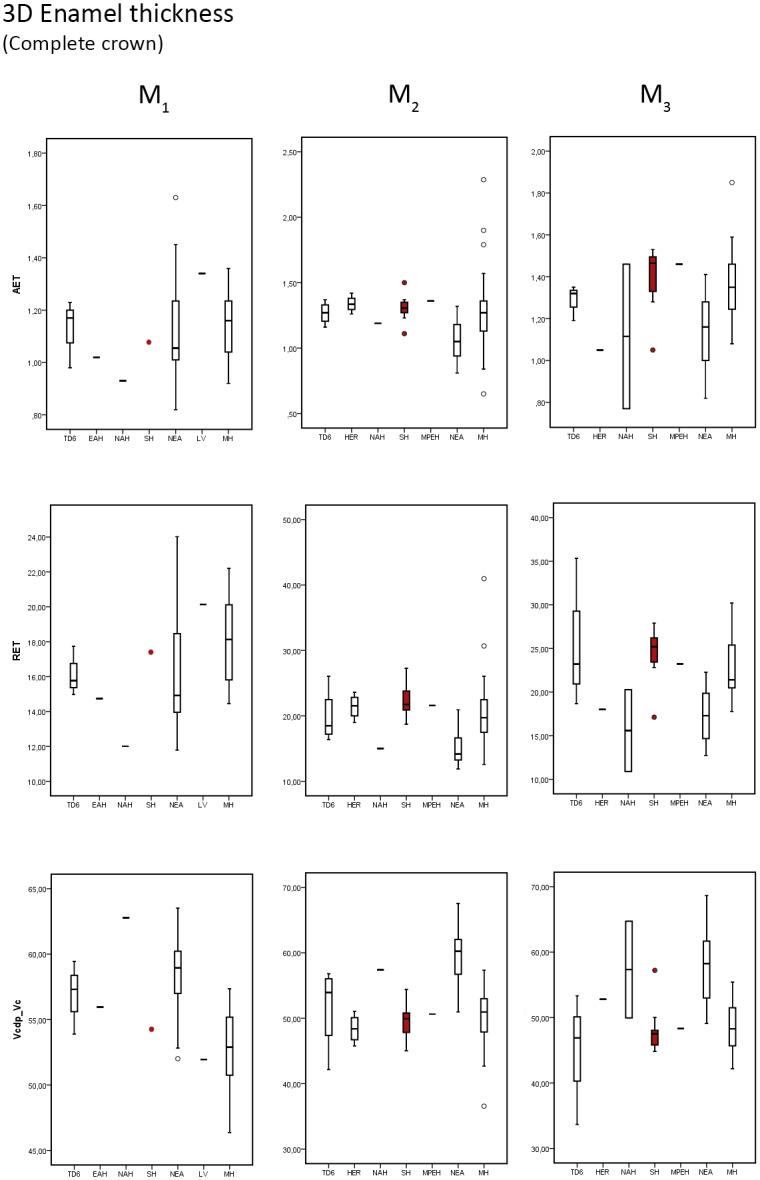
Box plots of the 3D, complete crown, values. 3D values depicting the average enamel thickness (3D AET), relative enamel thickness (3D RET) and the percentage of dentine and pulp in the total crown volume (Vcdp/Vc), in the mandibular molars of the SH and the comparative specimens/samples.

The SH mandibular molars (Table 4) also approximates the condition seen in TD6 [41], the European Middle Pleistocene specimen from Montmaurin-La Niche [53], and modern humans [25, 41, 55 and original data] and differs from that of Neanderthals [25, 28]. The SH lower mandibular values are within the range of variation of TD6, Neanderthal and modern humans variability, although they are close to the mean values of modern human estimates (Table 4 and Fig 5). When compared to the African Early Pleistocene *H*. *erectus/ergaster* isolated M_1_ from Mulhuli-Amo [52], the SH M_1_ shows a substantially higher 3D RET value. Similarly, the Middle Pleistocene specimens from the North African site of Tighenif [46] are outside the SH variability at all positions with thinner enamel. However, the generalised wear of Tighenif lower molars affect the enamel thickness estimates. On the contrary, the European Middle Pleistocene specimens from Montmaurin-La Niche [53] are within the SH variation range (Fig 5). Similarly, the Middle Pleistocene *H*. *erectus* M_2_ sample from Sangiran site [45] is within the SH range of variation. Finally, the European fossil *H*. *sapiens* from Lagar Velho (original data from NESPOS database) exceeds the SH M_1_ specimen (AT-829) 3D AET and 3D RET estimates (Table 4 and Fig 5).

**Fig 6:**
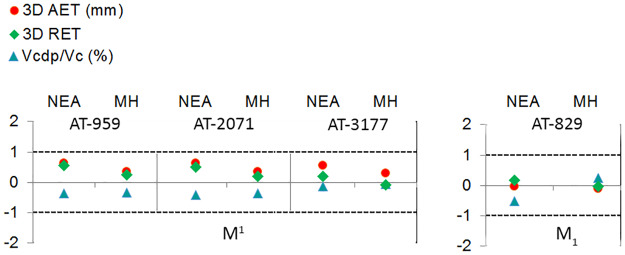
Adjusted Z score of the 3D variables (AET, RET and percentage of dentine). Assessed in the maxillary (specimens AT-959, AT-2071, AT-3177) and mandibular (specimen AT-829) molars from SH and compared to the variation expressed by Neanderthals (blue triangles) and modern humans (red circles). The solid line passing through zero represents the mean, and the other two lines correspond to the estimated 95% limit of variation expressed for the two comparative samples.

Fig 6 displays the results of the Adjusted Z-score test performed for the three upper and one lower M1 specimens from SH (AT-959, AT-2071, AT-3177 and AT-829, respectively) in comparison to the NEA and modern human populations for the three variables accounting for enamel thickness.

The complete, maxillary and mandibular molars, SH sample fall within the 95% of the variation of the NEA and MH groups for all the variables (3D AET, 3D RET and 3D Acdp/Ac). The SH specimens show closer relationship with the modern human population for the three variables than to the Neanderthals.

Table 5 shows the results of the Mann-Whitney statistical test between SH sample and comparative sample for the upper and lower M2s and M3s. For both upper and lower M2s and M3s, we solely observed statistical differences between SH and Neanderthals for the three variables (3D AET, 3D RET and 3D Vcdp/Vc). When comparing SH population with modern humans, we only identified statistical differences on the M^2^ for the RET variable.

**Table 5:** Mann-Whitney comparative statistical test (significant *p* values (0.05) in bold) for the 3D measurements (complete crown) in SH, and comparative samples (*H*. *antecessor*, *H*. *erectus*, Neanderthals and modern humans).

Groups	Tooth class	3D AET	3D RET	3D Vcdp/Vc
SH vs NEA	M^2^	**0.00**	**0.00**	**0.00**
SH vs MH		0.07	**0.02**	0.87
	M^3^			
SH vs NEA		**0.00**	**0.00**	**0.00**
SH vs MH		0.21	0.66	0.51
	M_2_			
SH vs TD6		0.62	0.15	0.20
SH vs HER		0.67	0.67	0.48
SH vs NEA		**0.00**	**0.00**	**0.00**
SH vs MH		0.42	0.53	0.28
	M_3_			
SH vs NEA		**0.00**	**0.00**	**0.00**
SH vs MH		0.13	0.11	0.58

Upper molars. SH: Atapuerca-Sima de los Huesos (original data); NEA: Neanderthals from various sites [25, 28, 50]; MH: modern humans [25, 41 and original data]. Lower molars. TD6: *H*. *antecessor* from Atapuerca-Gran Dolina [41]; HER: *H*. *erectus* from Sangiran [45]; SH: Atapuerca-Sima de los Huesos (orginal data); NEA: Neanderthals from various sites [25, 28, 50]; MH: modern humans [25, 41 and original data].

### 3D Lateral enamel thickness

**Fig 7:**
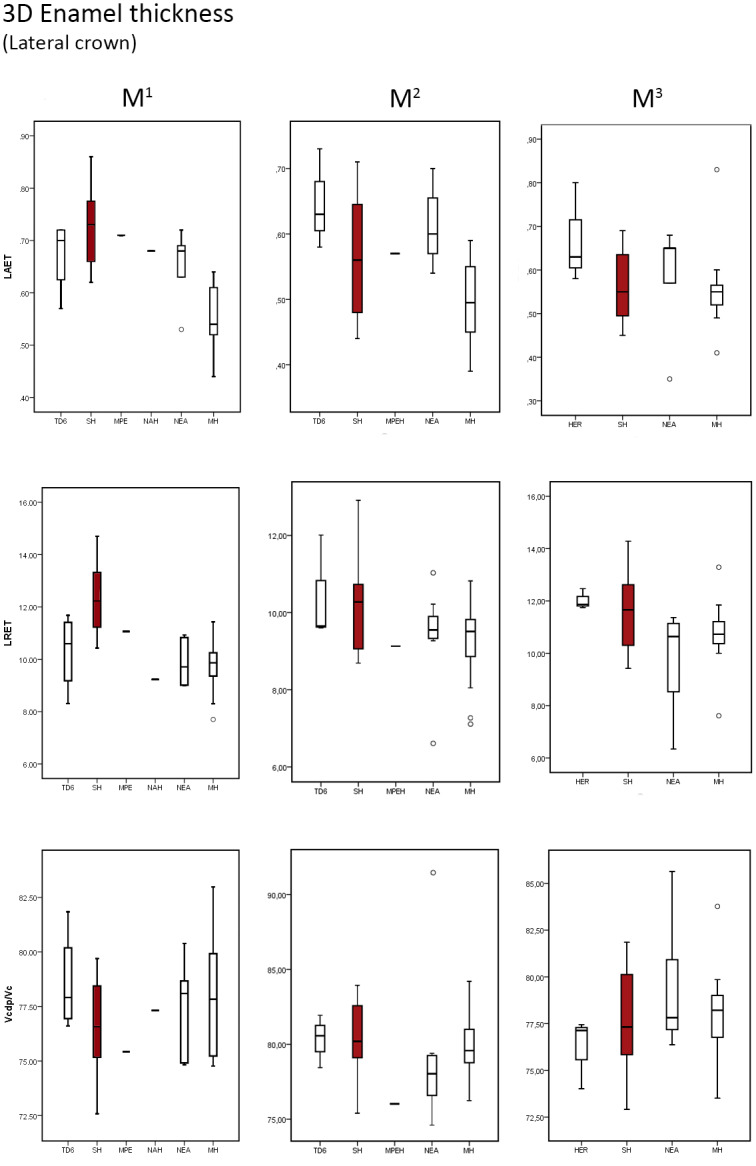
Box plots of the 3D, lateral enamel, values. 3D values depicting the lateral average enamel thickness (3D LAET), lateral relative enamel thickness (3D LRET) and the percentage of dentine and pulp in the lateral aspect of the crown (LVcdp/LVc), in the maxillary molars of the SH and the comparative specimens/samples.

**Fig 8:**
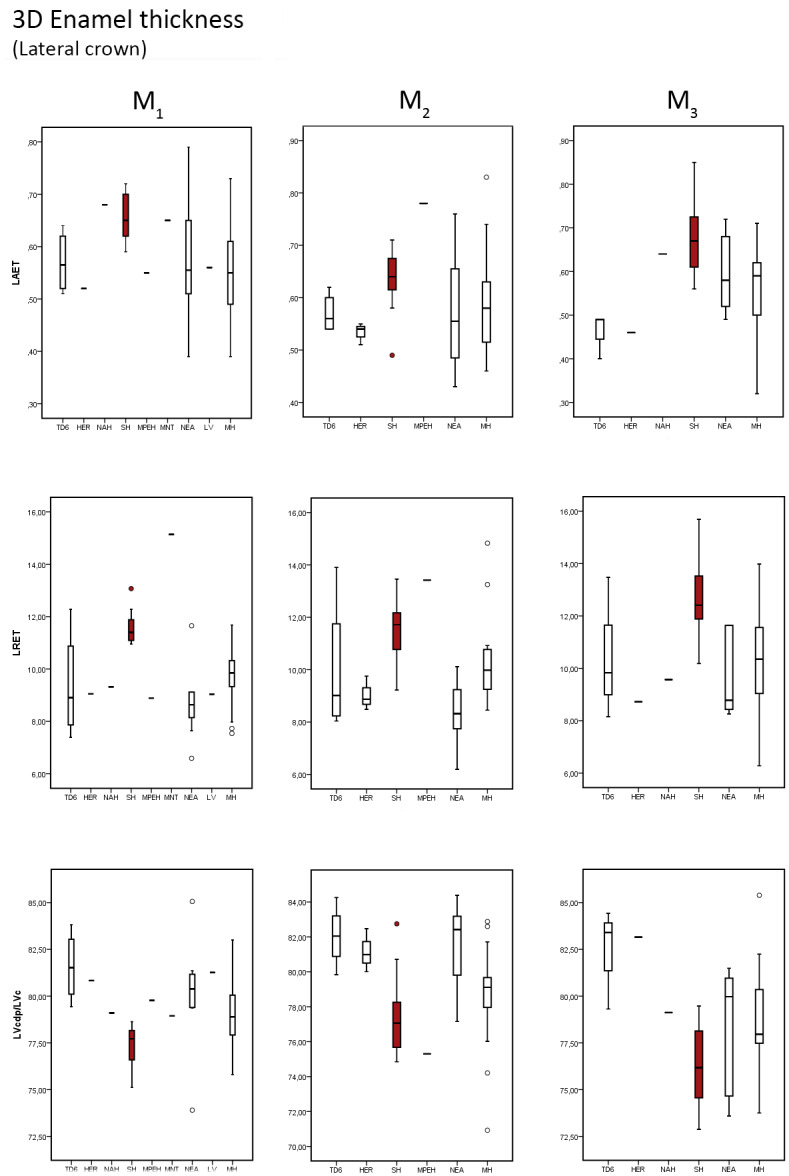
Box plots of the 3D, lateral enamel, values. 3D values depicting the lateral average enamel thickness (3D LAET), lateral relative enamel thickness (3D LRET) and the percentage of dentine and pulp in the lateral aspect of the crown (LVcdp/LVc), in the mandibular molars of the SH and the comparative specimens/samples.

Results of lateral tissue proportions in SH maxillary and mandibular revealed similar condition to what we described for the 2D and 3D crown proportions, although we also observe here differences with respect to modern humans (Table 6 and S3 Table for SH individual values, and Figs 7 and 8).

**Table 6:** 3D lateral enamel thickness variables assessed in SH maxillary and mandibular molars and compared with extinct and extant specimens/populations (SH data in bold). Mean and range are given for the comparative sample when having more than two specimens. Individual values are given for the rest of comparative sample.

Sample	N	Tooth class		3D LAET	3D LRET	VLcdp/VLc
TD6	4	M^1^	Mean	0.67	10.29	78.57
			SD	0.07	1.49	2.33
			Range	0.57–0.72	8.31–11.68	76.61–81.84
NAH_Tf	1			0.68	9.23	77.32
**SH**	**8**		**Mean**	**0.72**	**12.33**	**76.58**
			**SD**	**0.08**	**1.44**	**2.37**
			**Range**	**0.62–0.86**	**10.43–14.70**	**72.58–79.70**
MPEH_Vg	1			0.71	11.06	75.42
NEA	10		Mean	0.66	9.86	77.49
			SD	0.07	0.85	2.21
			Range	0.53–0.72	8.99–10.93	74.82–80.39
MH	14		Mean	0.55	9.75	78.03
			SD	0.06	0.97	2.54
			Range	0.44–0.64	7.70–11.43	74.77–82.98
		M^2^				
TD6	3		Mean	0.65	10.42	80.32
			SD	0.08	1.38	1.76
			Range	0.58–0.73	9.60–12.01	78.44–81.94
EMPH_Vg	1			0.57	9.13	76.03
**SH**	**12**		**Mean**	**0.56**	**10.16**	**80.47**
			**SD**	**0.09**	**1.26**	**2.49**
			**Range**	**0.44–0.71**	**8.69–12.91**	**75.40–83.94**
NEA	7		Mean	0.61	9.38	79.39
			SD	0.06	1.36	5.57
			Range	0.54–0.70	6.63–11.03	74.60–91.43
MH	14		Mean	0.50	9.18	79.97
			SD	0.06	1.08	2.22
			Range	0.39–0.59	7.11–10.82	76.24–84.20
		M^3^				
HER	3		Mean	0.67	12.03	76.20
			SD	0.11	0.39	1.90
			Range	0.58–0.80	11.75–12.47	74.01–77.45
**SH**	**15**		**Mean**	**0.56**	**11.59**	**77.58**
			**SD**	**0.08**	**1.53**	**2.88**
			**Range**	**0.45–0.69**	**9.42–14.28**	**72.91–81.85**
NEA	5		Mean	0.58	9.60	79.59
			SD	0.14	2.14	3.80
			Range	0.35–0.68	6.34–11.37	76.37–85.64
MH	12		Mean	0.56	10.73	78.10
			SD	0.10	1.31	2.48
			Range	0.41–0.83	7.65–13.29	73.51–83.77
		M_1_				
TD6	4		Mean	0.57	9.37	81.57
			SD	0.06	2.12	1.88
			Range	0.51–0.64	7.93–12.28	79.44–83.81
HER	1			0.52	9.05	80.83
NAH_Tig	1			0.68	9.32	79.10
MPEH_FR	1			0.55	8.89	79.77
**SH**	**13**		**Mean**	**0.66**	**11.56**	**77.33**
			**SD**	**0.04**	**0.63**	**1.07**
			**Range**	**0.59–0.72**	**10.95–13.07**	**75.11–78.63**
MPEH_M-LN	1			0.65	15.14	78.94
NEA	10		Mean	0.58	8.67	80.15
			SD	0.11	1.30	2.78
			Range	0.39–0.79	6.59–11.65	73.84–85.06
LV	1			0.56	9.04	81.26
MH	21		Mean	0.54	9.70	79.13
			SD	0.08	1.09	2.03
			Range	0.39–0.73	7.54–11.68	75.81–83.00
		M_2_				
TD6	4		Mean	0.57	9.99	82.05
			SD	0.04	2.69	1.8
			Range	0.54–0.62	8.04–13.90	79.83–84.25
HER	3		Mean	0.53	9.03	81.16
			SD	0.02	0.65	1.25
			Range	0.51–0.55	8.48–9.75	80.01–82.48
**SH**	**12**		**Mean**	**0.64**	**11.48**	**77.43**
			**SD**	**0.06**	**1.13**	**2.32**
			**Range**	**0.49–0.71**	**9.22–13.46**	**74.85–82.75**
MPEH_M-LN	1		2	0.78	13.42	75.3
NEA	8		Mean	0.57	8.36	81.54
			SD	0.11	1.23	2.43
			Range	0.43–0.76	6.19–10.11	77.15–84.38
MH	19		Mean	0.59	10.24	78.66
			SD	0.09	1.54	2.83
			Range	0.46–0.83	8.45–14.83	71.02–82.88
TD6	3	M_3_	Mean	0.46	10.48	82.38
			SD	0.05	2.72	2.71
			Range	0.40–0.49	8.15–13.47	79.31–84.43
HER	1			0.46	8.72	83.15
**SH**	**12**		**Mean**	**0.68**	**12.61**	**76.25**
			**SD**	**0.08**	**1.44**	**2.12**
			**Range**	**0.56–0.85**	**10.18–15.69**	**72.87–79.47**
NAH_Tig	1			0.64	9.57	79.12
MPEH_M-LN	1			0.77	12.96	75.28
NEA	6		Mean	0.59	9.59	78.44
			SD	0.09	1.61	3.42
			Range	0.49–0.72	8.26–11.65	73.59–81.49
MH	13		Mean	0.57	10.24	78.62
			SD	0.10	2.01	3.15
			Range	0.32–0.71	6.28–13.98	73.75–85.39

Upper molars: TD6: *H*. *antecessor* from Gran Dolina [41]. NAH: North African *Homo* (Tf: Tighenif [46]). SH: Atapuerca-Sima de los Huesos (original data). EMPH: European Middle Pleistocene *Homo* (Vg: Visogliano [24]). NEA: Neanderthals [41]. LV: Lagar Velho (Original data from Nespos). MH: modern humans [41 and original data].

Lower molars: TD6: *H*. *antecessor* from Gran Dolina [41]. NAH: North African *Homo* (Tf:Tighenif [46]). SH: Atapuerca-Sima de los Huesos (original data). HER: *H*. *erectus* (Sangiran, [45]). EMPH: European Middle Pleistocene *Homo* (FR: Fontana Ranuccio [24]). NEA: Neanderthals [41]. LV: Lagar Velho (Original data from Nespos). MH: modern humans [41 and original data].

The SH maxillary molars tend to display higher 3D LAET (Table 6) than the comparative sample, including Early Pleistocene *H*. *antecessor* [41], European and African Middle Pleistocene samples from Visiogliano [24] and Tighenif [46], Asian *H*. *erectus* [41], Neanderthals and modern humans [41 and original data], even though the SH results overlap with all of them. Once scaled through the 3D LRET, the differences are even more accentuated, as also illustrated by the low percentage of 3D LVcdp/LVc displayed by SH maxillary molars. The SH lower range values overlaps with the upper end of variation the TD6, Neanderthal and modern human variation (Fig 7). Compared with the European Middle Pleistocene specimens from Visogliano [24], the SH molars show similar 3D LRET. On the contrary, the African M^1^ from Tighenif [46] is outside the SH range, showing thinner enamel (Fig 7).

Similar results are found for the mandibular molars, although even more accentuated. The SH specimens show the highest mean values of 3D LRET (Table 6) of the comparative sample, overlapping with the maximal values or exceeding the estimates of the other comparative taxa except the European Middle Pleistocene specimen from Montmaurin-La Niche [53] that exhibits even higher 3D LRET than SH for the M_1_ and overlaps for the M_2_ (Fig 8). In turn, the SH mean values of 3D LVcdp/LVc are on average smaller than the comparative sample (Table 6 and Fig 8), even if overlapping with a few Neanderthals and modern humans [41 and original data], as well with the isolated molar from Tighenif [46] and with the mandibular molars from Montmaurin-La Niche [53].

Table 7 shows the results of the Mann-Whitney statistical test between SH sample and the comparative sample for the upper and lower molars.

**Table 7:** Mann-Whitney comparative statistical test (significant *p* values (0.05) in bold) for the 3D measurements (lateral enamel thickness) in SH, and comparative samples (*H*. *antecessor*, *H*. *erectus*, Neanderthals and modern humans).

Group	Tooth class	3D LAET	3D LRET	3D LVcdp/LVc
SH vs TD6	M^1^	0.34	0.08	0.23
SH vs NEA		0.15	**0.00**	0.69
SH vs MH		**0.00**	**0.00**	0.41
	M^2^			
SH vs NEA		0.23	0.55	0.10
SH vs MH		0.09	0.10	0.50
	M^3^			
SH vs NEA		0.38	0.10	0.31
SH vs MH		0.75	0.19	0.80
	M_1_			
SH vs TD6		**0.02**	0.10	**0.00**
SH vs NEA		**0.03**	**0.00**	**0.00**
SH vs MH		**0.00**	**0.00**	**0.00**
	M_2_			
SH vs TD6		**0.04**	0.18	**0.01**
SH vs NEA		0.18	**0.00**	**0.00**
SH vs MH		0.07	**0.00**	0.06
	M_3_			
SH vs NEA		0.11	**0.00**	0.13
SH vs MH		0.10	**0.00**	**0.05**

TD6: *H*. *antecessor* from Atapuerca-Gran Dolina [41]; SH: Atapuerca-Sima de los Huesos (original data); NEA: Neanderthals [41]; MH: modern humans [41 and original data].

As we detailed, the statistical analysis illustrates both the accentuated differences in terms of crown tissue proportions displayed by the SH mandibular molars with respect to the comparative samples, including with the modern humans.

In maxillary molars, differences are exclusively found in the M1 between SH and Neanderthals for the 3D LRET and between SH and modern humans for both 3D LAET and 3D LRET. On the contrary, in the lower molars, differences are seen between SH and TD6 for the 3D LAET and 3D LVcdp/LVc parameters, as well as between SH and Neanderthals and modern humans for the 3D LRET (and for 3D LAET and 3D LVcdp/LVc in the M_1_).

### Enamel thickness topographic distribution

**Fig 9:**
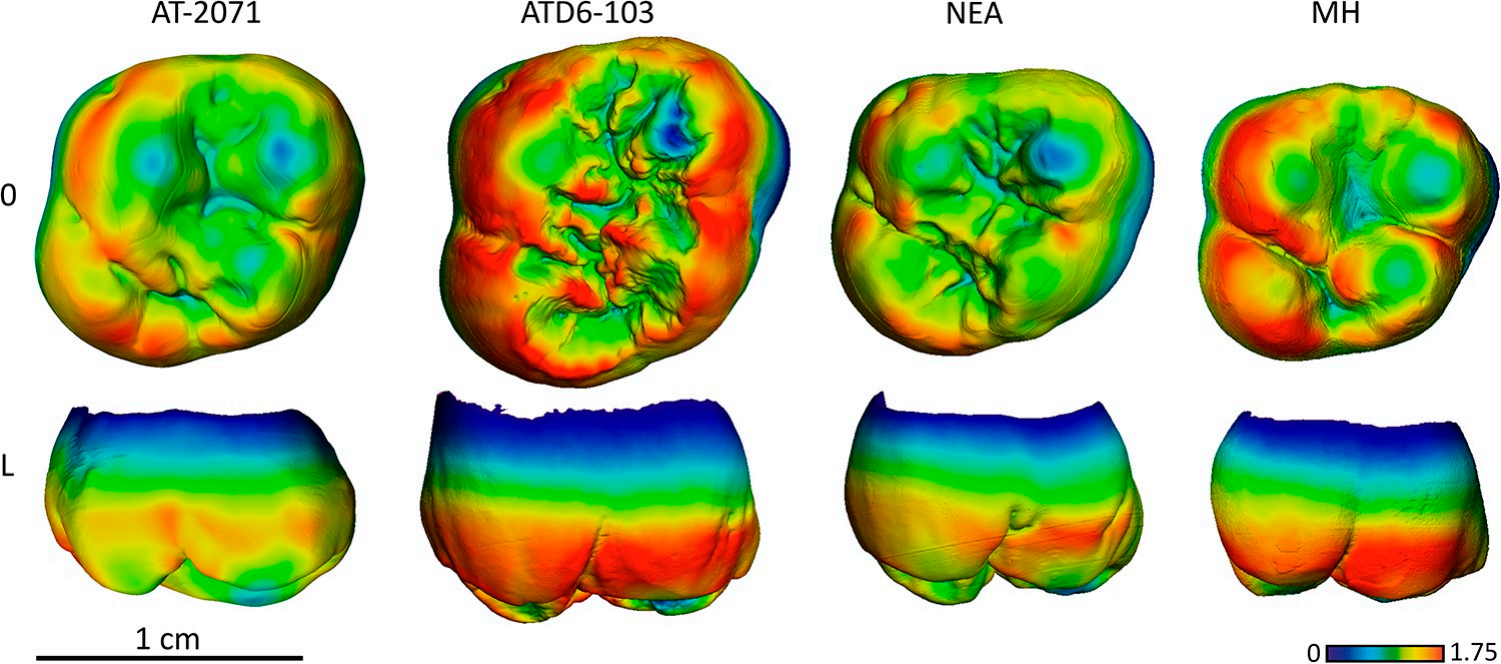
Enamel thickness cartographies of the Atapuerca-SH upper M1 (AT-2071) compared with those of *H*. *antecessor* (ATD6-103) from Atapuerca-Gran Dolina, Neanderthal and modern human. Topographic thickness variation is rendered by a pseudo-color scale ranging from thinner dark-blue to thicker red. NEA = Neanderthal (La Quina-H18) and MH = modern human of European origin (O = occlusal, L = lingual). Scale bar = 1.75 for all specimens. (When needed specimens have been mirrored to the left to match the SH specimen).

**Fig 10:**
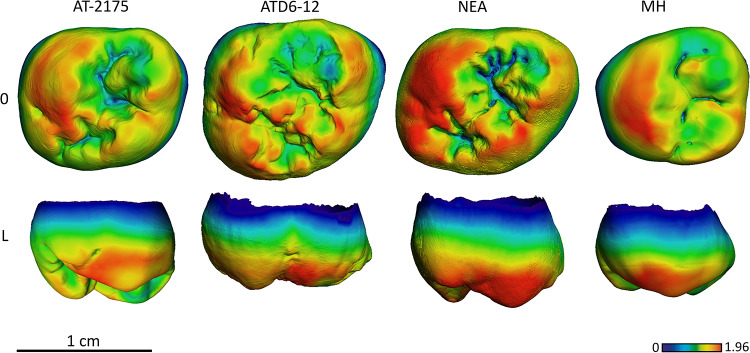
Enamel thickness cartographies of the Atapuerca-SH upper M2 (AT-2175) compared with those of *H*. *antecessor* (ATD6-12) from Atapuerca-Gran Dolina, Neanderthal and modern human. Topographic thickness variation is rendered by a pseudo-color scale ranging from thinner dark-blue to thicker red. NEA = Neanderthal (Krapina D96) and MH = modern human of European origin (O = occlusal, L = lingual). Scale bar = 1.96 for all specimens. (When needed specimens have been mirrored to the left to match the SH specimen).

**Fig 11:**
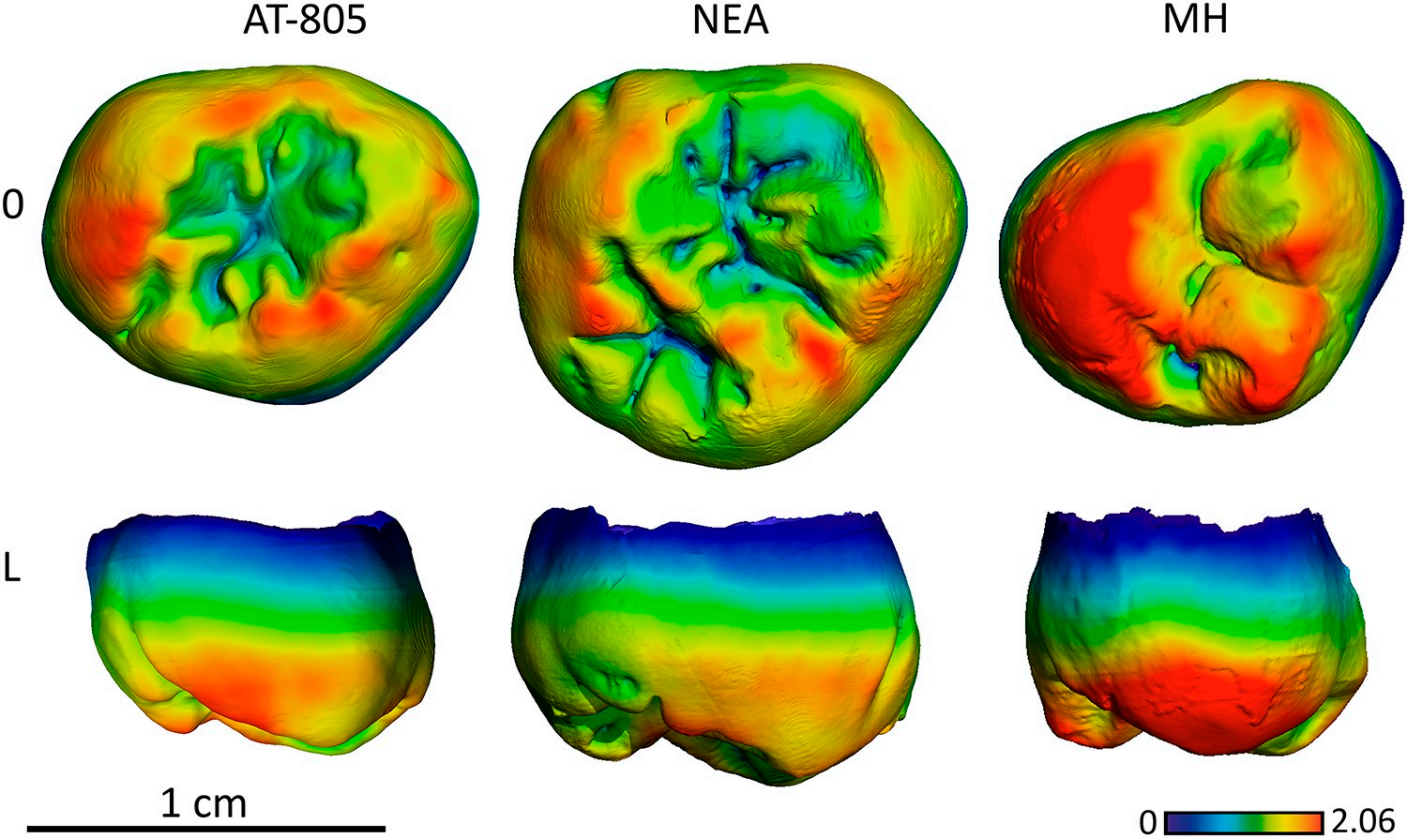
Enamel thickness cartographies of the Atapuerca-SH upper M3 (AT-805) compared with those of Neanderthal and modern human. Topographic thickness variation is rendered by a pseudo-color scale ranging from thinner dark-blue to thicker red. NEA = Neanderthal (Krapina, D99) and MH = modern human of European origin (O = occlusal, L = lingual). (When needed specimens have been mirrored to the left to match the SH specimen).

**Fig 12:**
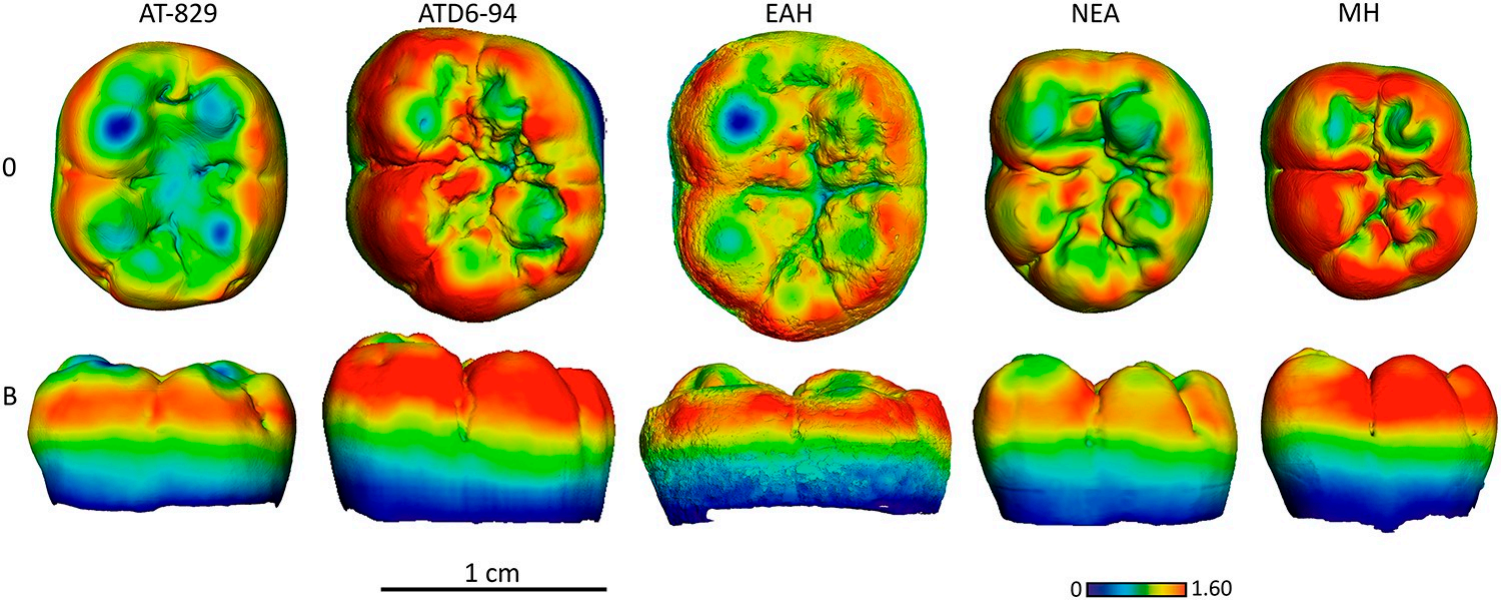
Enamel thickness cartographies of the Atapuerca-SH lower M1 (AT-829) compared with those of Mulhuli-Amo, *H*. *antecessor* (ATD6-94) from Atapuerca-Gran Dolina, Neanderthal and modern human. Topographic thickness variation is rendered by a pseudo-color scale ranging from thinner dark-blue to thicker red. EAH = MA93, NEA = Neanderthal (S14-7) and MH = modern human of European origin (O = occlusal, L = lingual). Scale bar = 1.60 for all specimens. (When needed specimens have been mirrored to the left to match the SH specimen).

**Fig 13:**
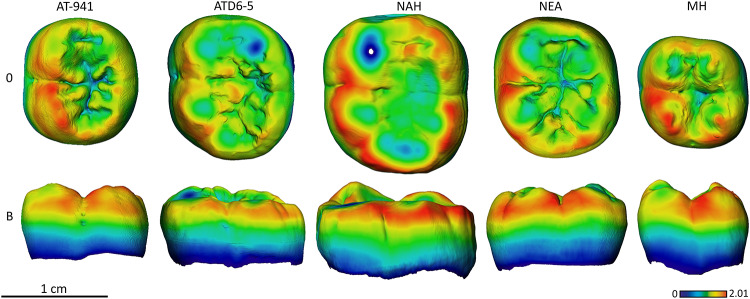
Enamel thickness cartographies of the Atapuerca-SH lower M2 (AT-941) compared with those of Tighenif, *H*. *antecessor* (ATD6-5) from Atapuerca-Gran Dolina, Neanderthal and modern human. Topographic thickness variation is rendered by a pseudo-color scale ranging from thinner dark-blue to thicker red. NAH = Tighenif, NEA = Neanderthal (Krapina, D10) and MH = modern human of European origin (O = occlusal, L = lingual). Scale bar = 1.60 for all specimens. (When needed specimens have been mirrored to the left to match the SH specimen).

**Fig 14:**
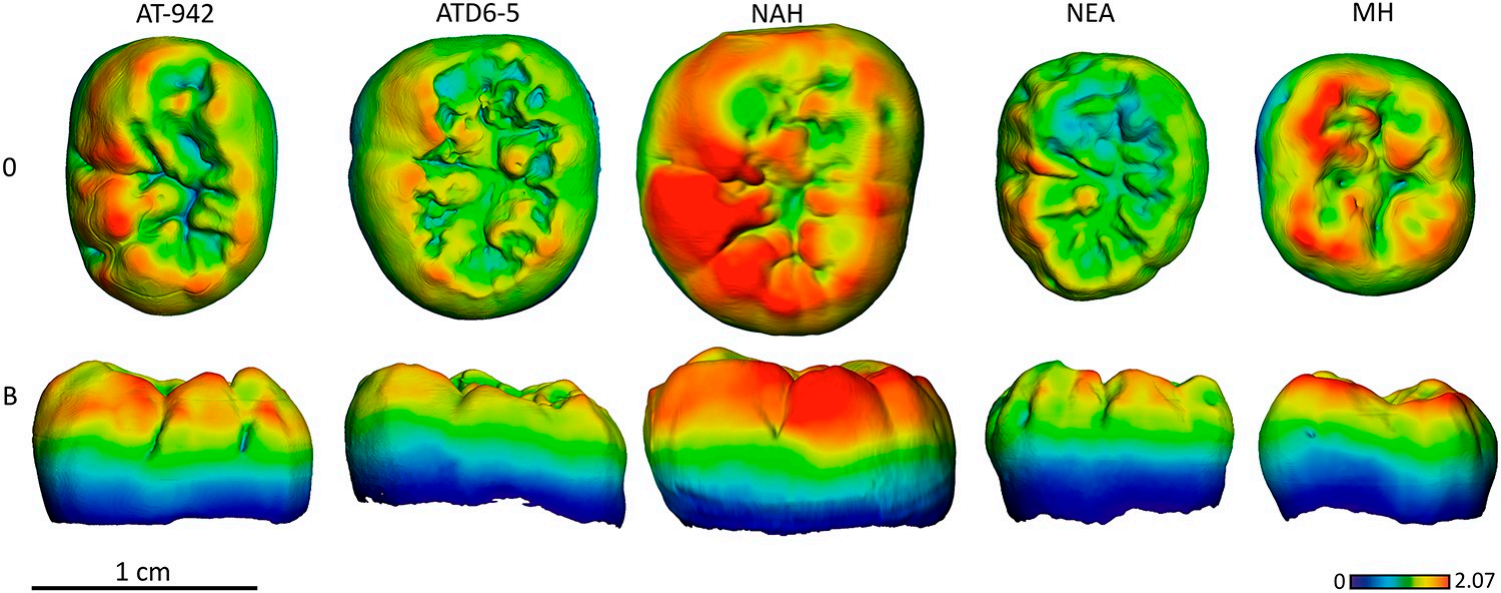
Enamel thickness cartographies of the Atapuerca-SH lower M3 (AT-942) compared with those of Tighenif, *H*. *antecessor* (ATD6-5) from Atapuerca-Gran Dolina, Neanderthal and modern human. Topographic thickness variation is rendered by a pseudo-color scale ranging from thinner dark-blue to thicker red. NAH = Tighenif, NEA = Neanderthal (Abri Suard S43) and MH = modern human of European origin (O = occlusal, L = lingual). Scale bar = 2.07 for all specimens. (When needed specimens have been mirrored to the left to match the SH specimen).

In general, the chromatic maps of SH molars approximates the Neanderthal signal in terms of enamel distribution among the crown (Figs 9–14).

The SH M^1^ approximate the Neanderthal condition in terms of absolute thickness and the pattern of enamel distribution, with thicker enamel on the lingual cusps and more peripherally distributed, compared to *H*. *antecessor* and modern humans (Fig 9).

The SH maxillary M^2^ approximate the TD6 figure in terms of absolute thickness, although shares with all the all comparative sample the relative enamel distribution, with thicker enamel on the lingual cusp(s) and buccal aspect of the paracone and metacone (Fig 10 and see S2 Fig. for more SH specimens).

As with the M^1^, the SH M^3^, as seen in specimen AT-805, approximates the Neanderthal condition both absolute thickness and distribution, with thicker enamel on the lingual cusps and more peripherally distributed, compared to modern humans (Fig 11 and see S3 Fig. for more SH specimens).

Despite the wear displayed by the SH M_1_, specimen AT-829, with regard to the relative distribution, the SH approximates the Neanderthal condition, with thicker enamel distributed at the external periphery of the buccal and lingual cusps and along the marginal edges (Fig 12), opposed to widely spread thickness over the occlusal basin seen in Early Pleistocene specimens from Atapuerca (specimen ATD6-94) and Eritrea (specimen MA93).

Although in terms of absolute enamel thickness, the SH M_2_ and M_3_ approximate the modern human condition, the pattern of distribution resembles that of *H*. *antecessor* and Neanderthals. The enamel is mostly distributed along the marginal edges, instead of the occlusal basin, and on the buccal and lingual aspects of the cusps. The SH M_2_ and M_3_ cartographic maps differ from the Middle Pleistocene specimen from Tighenif, as the latter shows thicker enamel on the lingual marginal ridges (Figs 13 and 14 and see S4 and S5 Figs for more SH specimens).

## Discussion

The European Middle Pleistocene human record is, with the exception of Sima de los Huesos (SH) and Caune de l’Arago assemblages [1, 15–17, 67], limited to isolated and chronospatially scattered remains [2, 68]. To date, the morphological studies of different localities across Europe suggest the existence of more than one hominin lineage [1, 15, 16, 24, 69] and a non-linear trajectory towards Neanderthals. This variability could be explained by discontinuous and intermittent hominin dispersals into Europe from an external source and a pattern of frequent isolation and fragmentation of these groups due to climatic instability [70–72]. Even if the evolutionary history of the Middle Pleistocene human groups remains controversial (e.g., [1, 2]), paleogenetic and morphological analyses demonstrated that the SH population is closely related to the Neanderthals [1, 18]. In fact, from a dental point of view, the morphology of the SH teeth is virtually undistinguishable from that of the Neanderthals [13–15, 17, 22, 73]. However, even if the external morphology is already Neanderthal-like in the SH population, the question is if the internal tooth structure also exhibits the condition that is typical to Neanderthals. That is, characterized by a proportionally thin enamel deposited over a larger volume of dentine in the molars [25, 28, 29, 33, 74].

Our analyses on the characterization of the SH molar enamel thickness show that this population exhibit the primitive condition, thick enamel, found in the *Homo* clade [25, 29, 62, 75]. The thick enamel shown by the SH molars is associated with a low percentage of dentine in the crown complex. Broadly, the SH molars approximate the condition documented in *H*. *antecessor* and *H*. *erectus*, as well as modern humans, as supported by our statistical analyses. To our knowledge, the primitive condition is shared by the majority of hominin species [25, 29, 41, 45, 51 and this study], except for Neanderthals [25, 28, 29, 31] and a few chronogeographically sparse specimens [52] showing relatively thinner molar enamel.

When considering intra-population or intra-species and dental class variability on crown enamel thickness, the Atapuerca (*H*. *antecessor*-TD6 and SH) populations are the only known samples displaying a combination of thin and thick enamelled teeth in the same dentitions, in contrast to what is already reported for Neanderthals and modern humans [25, 28, 29, 33]. More specifically, the TD6 and SH canines exhibit relatively thin enamel [42], while their molars have proportionally thick enamel [41 and this study].

Variation of enamel thickness in hominins probably results from the interplay of genetic [76], developmental and life history features [38–40, 77], the structural organization of the mineralized dental tissues [25, 37], dental and body size reduction [29, 78, 79 and references therein], and dietary adaptions (at least at the genus level [34, 80]). The unique pace of dental development in *H*. *sapiens*, slow trajectory of enamel growth combined with an initial slow rate of tooth root extension, together with the extreme reduction of the jaw and posterior dentition (i.e., involving allometric reduction of enamel and dentine volumes) could relate to the thick enamel pattern exhibited by this species [38–40].

Regarding the SH assemblage, metric estimates demonstrated that SH postcanine dentition has similar dimensions than those of modern humans [81] and smaller than Neanderthals [17]. The SH reduced molar dimensions was explained as the result of a decrease of the rate of cell proliferation, which affected the later-forming crown regions to a greater extent [81, 82]. However, although we could speculate with dental reduction as the main factor behind the thick enamel in SH population, the difference of cusp size variation between SH and modern humans suggests different evolutionary mechanisms of dental reduction in these groups [81, 83]. Similarly, the body mass estimates for the SH individuals [84], which is an indicator of the species robusticity, would not support the association between coronal dentine reduction and body gracility [29, 85 and references therein]. Altogether, this suggests that the mechanisms underlying the thick enamel in SH may be different from those driving the thick enamel in *H*. *sapiens*, and in the SH population it may be simply the retention of the plesiomorphic condition for the genus *Homo*. In this scenario, the typical thin pattern of Neanderthals in molars could be a late acquisition in the evolution of this lineage. However, until more data is available on the enamel thickness in other Early and Middle Pleistocene groups this scenario remains as a tentative hypothesis.

## Conclusion

In this study, we provide the data on the dental tissue proportions and enamel thickness in the molar collection belonging to the Middle Pleistocene SH population, and contribute to the characterisation of the variability of this trait in the genus *Homo*. For the complete crown measurements, SH molars tend to show on average 2D and 3D absolute and relative thick enamel, corresponding to the plesiomorphic condition seen in the majority of the fossil sample and modern humans, and in contrast to the Neanderthal condition. Moreover, the SH lateral enamel thickness is thicker compared to both Neanderthals and modern humans, highlighting the absolute contribution of the lateral surface to the overall thick enamel distribution on the crown. On the contrary, the relative pattern of occlusal enamel distribution of the SH molar assemblage resembles the Neanderthal condition. Due to the phylogenetic position of the SH population, the thick condition in molars could represent the persistence of the plesiomorphic condition in this group. However, more data is needed on other Early and Middle Pleistocene populations to fully understand the evolutionary meaning of this trait. To our knowledge, the Atapuerca SH group is the only population of the genus *Homo* to exhibit a combination of the primitive (molars) and derived (canines) condition for the enamel thickness trait [41, 42]. Future studies of the complete dentition will provide a more comprehensive understanding of the anterior versus posterior enamel thickness pattern in the populations of the Early and Middle Pleistocene in Europe and its evolutionary interpretation.

## Supporting information

S1 Fig Virtual extraction of sections. A. Surface of the molar crown. B. Identification (red ring) of the main dentine horns scrolling through the image stack. C. Re-sliced image showing the tips of themain dentine horns (paracone, protocone and metacone in upper molars, and protoconid, metaconid and hypoconid in lower molars) used for the reference plane (within the red ring). D. Positioning of the buccolingual section, perpendicular to the reference plane and passing through the dentine horn tips of the mesial cusps. E. Virtual buccolingual section where measurements will be acquired.
(TIF)

S2 Fig Enamel thickness cartographies of the Atapuerca-SH upper M2s (AT-2175, AT-824, and AT-960) compared with those of *H*. *antecessor* (ATD6-12) from Atapuerca-Gran Dolina, Neanderthal and modern human. Topographic thickness variation is rendered by a pseudo-color scale ranging from thinner dark-blue to thicker red. NEA = Neanderthal (Krapina D96) and MH = modern human of European origin (O = occlusal, L = lingual). Scale bar = 1.96 for all specimens. (When needed specimens have been mirrored to the left to match the SH specimen).
(TIF)

S3 Fig Enamel thickness cartographies of the Atapuerca-SH upper M3s (AT-805, AT-5292, AT-3181, and AT-274) compared with those of Neanderthal and modern human. Topographic thickness variation is rendered by a pseudo-color scale ranging from thinner dark-blue to thicker red. NEA = Neanderthal (Krapina, D99) and MH = modern human of European origin (O = occlusal, L = lingual). (When needed specimens have been mirrored to the left to match the SH specimen).
(TIF)

S4 Fig Enamel thickness cartographies of the Atapuerca-SH lower M2s (AT-941, AT-2396, and AT-6579) compared with those of *H*. *antecessor* (ATD6-5) from Atapuerca-Gran Dolina, Tighenif, Neanderthal and modern human. Topographic thickness variation is rendered by a pseudo-color scale ranging from thinner dark-blue to thicker red. NAH = Tighenif, NEA = Neanderthal (Krapina, D10) and MH = modern human of European origin (O = occlusal, L = lingual). Scale bar = 1.60 for all specimens. (When needed specimens have been mirrored to the left to match the SH specimen).
(TIF)

S5 Fig Enamel thickness cartographies of the Atapuerca-SH lower M3s (AT-942, AT-3182, AT-1468, and AT-2777) compared with those of *H*. *antecessor* (ATD6-5) from Atapuerca-Gran Dolina, Tighenif, Neanderthal and modern human. Topographic thickness variation is rendered by a pseudo-color scale ranging from thinner dark-blue to thicker red. NAH = Tighenif, NEA = Neanderthal (Abri Suard S43) and MH = modern human of European origin (O = occlusal, L = lingual). Scale bar = 2.07 for all specimens. (When needed specimens have been mirrored to the left to match the SH specimen).
(TIF)

S1 Table 2D values measured in the SH maxillary and mandibular molars and those of the extinct and extant specimens/populations. (DOCX)

S2 Table 3D enamel thickness, complete crown, values measured in the SH maxillary and mandibular molars and those of the extinct and extant specimens/populations. (DOCX)

S3 Table 3D lateral enamel thickness values measured in the SH maxillary and mandibular molars and those of the extinct and extant specimens/populations. (DOCX)

S1 Data (DOCX)
