# Supplementary figures and images for: Crown tissue proportions and enamel thickness distribution in the Middle Pleistocene hominin molars from Sima de los Huesos (SH) population (Atapuerca, Spain)
Source: PLoS One. 2020 Jun 8;15(6):e0233281. doi: 10.1371/journal.pone.0233281 (PMC7279586; doi:10.1371/journal.pone.0233281)

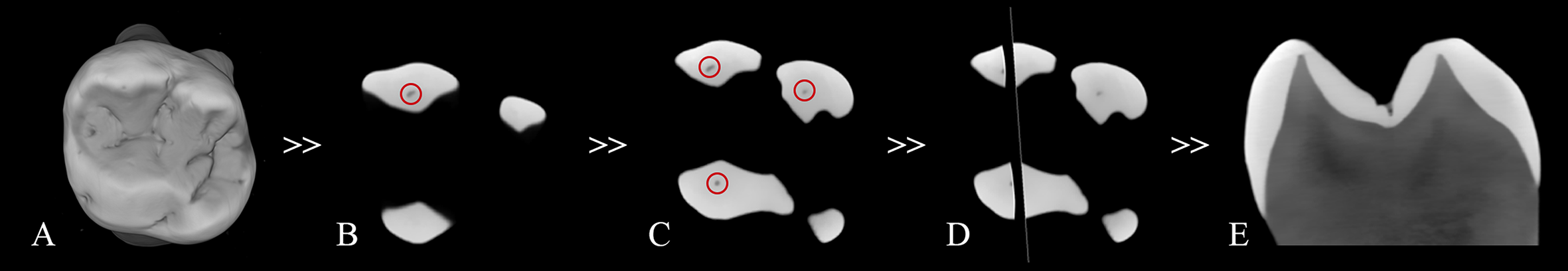

Supplement: S1 Fig — A. Surface of the molar crown. B. Identification (red ring) of the main dentine horns scrolling through the image stack. C. Re-sliced image showing the tips of themain dentine horns (paracone, protocone and metacone in upper molars, and protoconid, metaconid and hypoconid in lower molars) used for the reference plane (within the red ring). D. Positioning of the buccolingual section, perpendicular to the reference plane and passing through the dentine horn tips of the mesial cusps. E. Virtual buccolingual section where measurements will be acquired. (TIF) [file pone.0233281.s001.tif]

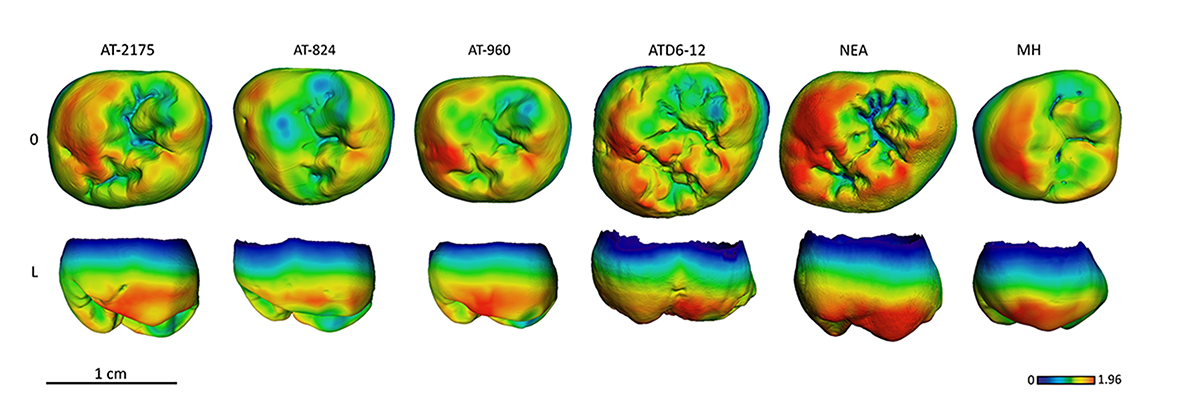

Supplement: S2 Fig — Topographic thickness variation is rendered by a pseudo-color scale ranging from thinner dark-blue to thicker red. NEA = Neanderthal (Krapina D96) and MH = modern human of European origin (O = occlusal, L = lingual). Scale bar = 1.96 for all specimens. (When needed specimens have been mirrored to the left to match the SH specimen). (TIF) [file pone.0233281.s002.tif]

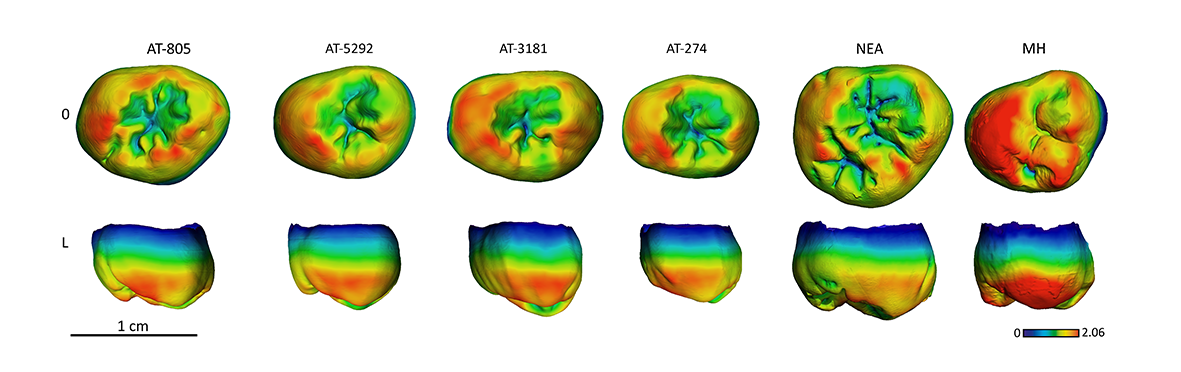

Supplement: S3 Fig — Topographic thickness variation is rendered by a pseudo-color scale ranging from thinner dark-blue to thicker red. NEA = Neanderthal (Krapina, D99) and MH = modern human of European origin (O = occlusal, L = lingual). (When needed specimens have been mirrored to the left to match the SH specimen). (TIF) [file pone.0233281.s003.tif]

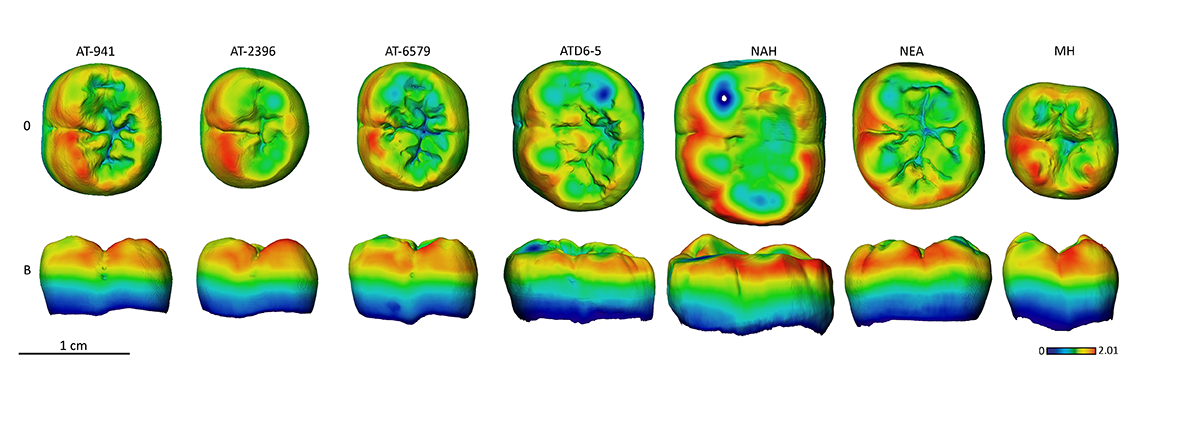

Supplement: S4 Fig — Topographic thickness variation is rendered by a pseudo-color scale ranging from thinner dark-blue to thicker red. NAH = Tighenif, NEA = Neanderthal (Krapina, D10) and MH = modern human of European origin (O = occlusal, L = lingual). Scale bar = 1.60 for all specimens. (When needed specimens have been mirrored to the left to match the SH specimen). (TIF) [file pone.0233281.s004.tif]

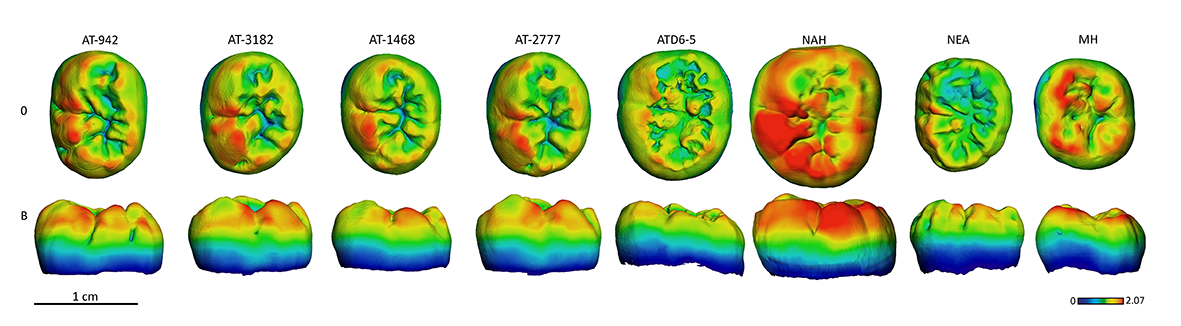

Supplement: S5 Fig — Topographic thickness variation is rendered by a pseudo-color scale ranging from thinner dark-blue to thicker red. NAH = Tighenif, NEA = Neanderthal (Abri Suard S43) and MH = modern human of European origin (O = occlusal, L = lingual). Scale bar = 2.07 for all specimens. (When needed specimens have been mirrored to the left to match the SH specimen). (TIF) [file pone.0233281.s005.tif]
