# Supplementary material for: Crown tissue proportions and enamel thickness distribution in the Middle Pleistocene hominin molars from Sima de los Huesos (SH) population (Atapuerca, Spain)
Source: PLoS One. 2020 Jun 8;15(6):e0233281. doi: 10.1371/journal.pone.0233281 (PMC7279586; doi:10.1371/journal.pone.0233281)
Supplement: S1 Table — (DOCX) [file pone.0233281.s006.docx]

S1 Table. 2D values measured in the SH maxillary and mandibular molars and those of the extinct and extant specimens/populations.

| Sample | N | Tooth class |  | c (mm2) | b (mm2) | a(mm2) | e (mm) | a/b*100 | AET (mm) | RET |
| --- | --- | --- | --- | --- | --- | --- | --- | --- | --- | --- |
| TD6 | 4 | M^1^ | Mean | 23.01 | 42.8 | 65.81 | 20.57 | 64.98 | 1.12 | 17.14 |
|  |  |  | SD | 1.84 | 4.23 | 5.22 | 1.23 | 2.4 | 0.06 | 1.15 |
|  |  |  | Range | 20.52-24.56 | 37.33-47.66 | 60.05-72.22 | 19.24-22.14 | 62.16-67.72 | 1.07-1.21 | 16.07-18.38 |
| AT-599 |  |  |  | 20.88 | 36.01 | 56.89 | 20.22 | 63.30 | 1.03 | 17.21 |
| AT-2071 |  |  |  | 21.94 | 37.05 | 58.99 | 21.13 | 62.81 | 1.04 | 17.06 |
| AT-3177 |  |  |  | 23.25 | 43.88 | 67.13 | 21.90 | 65.37 | 1.06 | 16.03 |
| **SH** | **3** |  | **Mean** | **22.02** | **38.98** | **61** | **21.08** | **63.82** | **1.04** | **16.76** |
|  |  |  | **SD** | **1.19** | **4.28** | **5.41** | **0.84** | **1.36** | **0.02** | **0.64** |
|  |  |  | **Range** | **20.88-23.25** | **36.01-43.88** | **56.89-67.13** | **20.22-21.90** | **62.81-65.37** | **1.03-1.06** | **16.03-17.21** |
| HER | 2 |  | Mean | 30.04 | 49.52 | 79.55 | 22.88 | 62.22 | 1.31 | 18.67 |
|  |  |  | SD | 1.61 | 4.12 | 5.73 | 0.95 | 0.7 | 0.02 | 0.56 |
|  |  |  | Range | 28.90-31.17 | 46.60-52.43 | 75.50-83.60 | 22.20-23.55 | 61.72-62.72 | 1.30-1.32 | 18.28-19.07 |
| MPEH_St | 1 |  |  | 23.25 | 40.99 | 64.24 | 21.49 | 63.81 | 1.08 | 16.9 |
| NEA | 5 |  | Mean | 22.98 | 43.94 | 66.92 | 22.36 | 65.68 | 1.03 | 15.50 |
|  |  |  | SD | 2.95 | 4.55 | 7.06 | 1.06 | 1.88 | 0.10 | 1.22 |
|  |  |  | Range | 21.03-28.00 | 37.19-49.75 | 58.22-77.75 | 21.08-23.45 | 63.88-68.04 | 0.93-1.19 | 13.80-16.93 |
| *MH | 37 |  | Mean | 42.87 | 25.18 | 68.05 | 20.64 | 62.85 | 1.22 | 18.75 |
|  |  |  | SD | 6.25 | 3.16 |  | 1.51 | 2.67 | 0.12 | 2.08 |
|  |  |  | Range | 32.46-59.44 | 20.05-31.82 |  | 17.66-24.11 | 57.16-68.98 | 0.98-1.50 | 13.95-23.86 |
| MH | 12 |  | Mean | 20.44 | 34.73 | 55.18 | 19.11 | 63.11 | 1.07 | 18.13 |
|  |  |  | SD | 3.52 | 2.73 | 5.71 | 0.61 | 3.06 | 0.18 | 2.66 |
|  |  |  | Range | 16.54-28.50 | 30.77-41.00 | 48.94-69.50 | 17.70-19.82 | 58.31-67.58 | 0.84-1.45 | 14.46-22.71 |
|  |  | M^2^ |  |  |  |  |  |  |  |  |
| TD6 | 2 |  | Mean | 26.66 | 40.94 | 67.6 | 19.13 | 60.65 | 1.39 | 21.74 |
|  |  |  | SD | 3.78 | 2.67 | 6.45 | 0.25 | 1.83 | 0.18 | 2.09 |
|  |  |  | Range | 28.90-31.17 | 39.05-42.83 | 63.04-72.16 | 18.95-19.30 | 59.35-61.94 | 1.27-1.52 | 20.26-23.22 |
| AT-12 |  |  |  | 20.12 | 31.65 | 51.77 | 19.02 | 61.14 | 1.06 | 18.80 |
| AT-824 |  |  |  | 25.48 | 37.02 | 62.50 | 20.31 | 59.23 | 1.25 | 20.62 |
| AT-15 |  |  |  | 26.05 | 43.91 | 69.96 | 21.56 | 62.76 | 1.21 | 18.23 |
| AT-170 |  |  |  | 23.44 | 37.48 | 60.92 | 19.58 | 61.52 | 1.20 | 19.55 |
| AT-960 |  |  |  | 25.64 | 33.12 | 58.76 | 20.05 | 56.36 | 1.28 | 22.22 |
| AT-822 |  |  |  | 22.29 | 41.24 | 63.53 | 20.56 | 64.91 | 1.08 | 16.88 |
| AT-2175 |  |  |  | 25.92 | 36.44 | 62.36 | 20.37 | 58.43 | 1.27 | 21.08 |
| AT-6215 |  |  |  | 19.98 | 29.96 | 49.94 | 18.644 | 59.99 | 1.07 | 19.58 |
| **SH** | **8** |  | **Mean** | **23.62** | **36.35** | **59.97** | **20.01** | **60.55** | **1.18** | **19.62** |
|  |  |  | **SD** | **2.56** | **4.72** | **6.49** | **0.92** | **2.65** | **0.09** | **1.69** |
|  |  |  | **Range** | **19.98-26.05** | **29.96-43.91** | **49.94-69.96** | **18.64-21.56** | **56.36-64.91** | **1.06-1.28** | **16.88-22.22** |
| HER | 2 |  | Mean |  |  |  |  |  | 1.49 | 21.47 |
|  |  |  | SD |  |  |  |  |  | 0.02 | 2.9 |
|  |  |  | Range |  |  |  |  |  | 1.48-1.51 | 19.42-23.52 |
| MPEH_St | 1 |  |  | 25.79 | 48.99 | 74.78 | 21.51 | 65.51 | 1.2 | 17.13 |
| MPAH_TQ | 1 |  |  | 32.02 | 56.78 | 88.8 | 22.59 | 63.94 | 1.42 | 18.81 |
| NEA | 6 |  | Mean | 25.99 | 44.53 | 70.51 | 21.65 | 62.96 | 1.2 | 18.12 |
|  |  |  | SD | 2.61 | 7.52 | 9.45 | 1.57 | 2.75 | 0.08 | 1.84 |
|  |  |  | Range | 22.72-29.05 | 38.55-58.72 | 62.59-87.77 | 20.03-24.22 | 59.17-66.90 | 1.13-1.29 | 15.65-20.85 |
| FHS_Qz |  |  |  | 25.97 | 43.63 | 69.6 | 19.82 | 62.69 | 1.31 | 19.84 |
| *MH | 25 |  | Mean | 42.76 | 28.61 | 71.37 | 20.49 | 60.05 | 1.4 | 21.59 |
|  |  |  | SD | 7.9 | 4.07 |  | 1.67 | 3.46 | 0.17 | 3.13 |
|  |  |  | Range | 30.12-65.71 | 23.12-36.64 |  | 18.22-24.83 | 53.80-66.45 | 1.13-1.76 | 16.49-28.03 |
| MH | 12 |  | Mean | 25.36 | 36.95 | 62.31 | 19.28 | 59.59 | 1.32 | 21.75 |
|  |  |  | SD | 5.07 | 2.99 | 5.34 | 0.62 | 5.71 | 0.27 | 4.83 |
|  |  |  | Range | 15.20-34.30 | 30.90-42.21 | 50.70-71.30 | 18.51-20.71 | 49.69-70.02 | 0.82-1.80 | 13.72-29.59 |
|  |  | M^3^ |  |  |  |  |  |  |  |  |
| AT-10 |  |  |  | 22.00 | 32.89 | 54.89 | 18.19 | 59.92 | 1.21 | 21.09 |
| AT-194 |  |  |  | 26.58 | 36.93 | 63.51 | 19.48 | 58.15 | 1.36 | 22.45 |
| AT-601 |  |  |  | 30.30 | 30.08 | 60.38 | 21.64 | 49.82 | 1.40 | 25.53 |
| AT-805 |  |  |  | 27.31 | 33.56 | 60.87 | 18.93 | 55.13 | 1.44 | 24.90 |
| AT-826 |  |  |  | 19.45 | 25.45 | 44.90 | 17.29 | 56.68 | 1.12 | 22.30 |
| AT-3181 |  |  |  | 28.23 | 32.02 | 60.25 | 19.75 | 53.15 | 1.43 | 25.26 |
| AT-1471 |  |  |  | 23.36 | 21.12 | 44.48 | 19.17 | 47.48 | 1.22 | 26.52 |
| AT-2393 |  |  |  | 21.49 | 28.63 | 50.12 | 17.53 | 57.12 | 1.23 | 22.91 |
| AT-3183 |  |  |  | 22.05 | 27.38 | 49.43 | 17.72 | 55.39 | 1.24 | 23.78 |
| AT-5082 |  |  |  | 30.24 | 28.23 | 58.47 | 20.65 | 48.28 | 1.46 | 27.56 |
| AT-5292 |  |  |  | 26.54 | 31.82 | 58.36 | 19.76 | 54.52 | 1.34 | 23.81 |
| AT-274 |  |  |  | 21.29 | 27.23 | 48.52 | 20.52 | 56.12 | 1.04 | 19.88 |
| AT-602 |  |  |  | 28.34 | 35.94 | 64.28 | 20.07 | 55.91 | 1.41 | 23.55 |
| AT-615 |  |  |  | 22.21 | 27.32 | 49.53 | 17.51 | 55.16 | 1.27 | 24.27 |
| **SH** | **14** |  | **Mean** | **24.96** | **29.9** | **54.86** | **19.16** | **54.49** | **1.3** | **23.84** |
|  |  |  | **SD** | **3.64** | **4.28** | **6.86** | **1.35** | **3.64** | **0.13** | **2.07** |
|  |  |  | **Range** | **19.45-30.30** | **21.12-36.93** | **44.48-64.28** | **17.29-21.64** | **47.48-59.92** | **1.04-1.46** | **19.88-27.56** |
| MPEH_St | 1 |  |  | 22.02 | 33.35 | 55.37 | 17.66 | 60.23 | 1.25 | 21.59 |
| MPAH_TQ | 1 |  |  | 31.44 | 46.17 | 77.61 | 20.15 | 59.49 | 1.56 | 22.96 |
| NEA | 6 |  | Mean | 22.83 | 44.56 | 67.39 | 20.54 | 66.4 | 1.1 | 16.45 |
|  |  |  | SD | 5.89 | 8.22 | 13.77 | 1.89 | 2.88 | 0.19 | 1.94 |
|  |  |  | Range | 14.09-32.00 | 33.00-57.68 | 47.09-89.68 | 17.75-23.62 | 63.51-70.08 | 0.79-1.35 | 13.82-18.49 |
| *MH | 52 |  | Mean | 40.97 | 27.20 |  | 19.72 | 59.93 | 1.38 | 21.75 |
|  |  |  | SD | 7.46 | 3.58 |  | 1.7 | 3.23 | 0.14 | 2.85 |
|  |  |  | Range | 27.39-55.87 | 18.91-38.39 |  | 16.02-23.15 | 52.35-66.13 | 1.18-1.95 | 17.02-30.01 |
| MH | 11 |  | Mean | 23.62 | 34.46 | 58.08 | 18.16 | 59.17 | 1.30 | 22.38 |
|  |  |  | SD | 3.68 | 4.45 | 4.74 | 0.90 | 5.57 | 0.20 | 4.10 |
|  |  |  | Range | 16.80-28.99 | 30.31-45.60 | 51.89-68.40 | 16.70-19.80 | 52.58-68.54 | 1.01-1.64 | 16.63-28.99 |
|  |  | M_1_ |  | c (mm2) | b (mm2) | a(mm2) | e (mm) | a/b*100 | AET (mm) | RET |
| TD6 | 4 |  | Mean | 22.05 | 35.27 | 57.31 | 18.86 | 61.25 | 1.17 | 19.92 |
|  |  |  | SD | 2.93 | 7.17 | 8.50 | 1.13 | 5.03 | 0.16 | 3.26 |
|  |  |  | Range | 18.07-25.02 | 27.35-44.67 | 49.33-67.79 | 18.00-20.47 | 55.44-65.89 | 1.00-1.38 | 16.90-23.12 |
| EAH | 1 |  |  | 18.6 | 38.2 | 56.8 | 18.6 | 67.25 | 1 | 16.17 |
| **AT-829** | **1** |  |  | **20.33** | **31.16** | **51.49** | **20.27** | **60.51** | **1.00** | **17.96** |
| NEA | 13 |  | Mean | 21.12 | 40.44 | 61.56 | 21.07 | 65.62 | 1.00 | 15.88 |
|  |  |  | SD | 1.70 | 3.90 | 4.54 | 1.83 | 2.59 | 0.07 | 1.69 |
|  |  |  | Range | 16.82-23.81 | 33.38-45.42 | 51.42-66.07 | 17.65-23.25 | 60.49-69.28 | 0.92-1.18 | 13.77-20.46 |
| *MH | 55 |  | Mean | 40.16 | 21.74 | 61.9 | 20.32 | 64.48 | 1.07 | 16.99 |
|  |  |  | SD | 5.02 | 2.95 |  | 1.28 | 3.073 | 0.13 | 2.29 |
|  |  |  | Range | 27.45-50.82 | 16.21-28.58 |  | 16.73-22.94 | 59.19-72.65 | 0.80-1.40 | 11.76-22.62 |
| MH | 11 |  | Mean | 20.94 | 32.97 | 53.91 | 18.83 | 61.09 | 1.11 | 19.47 |
|  |  |  | SD | 1.81 | 3.44 | 4.60 | 1.16 | 2.24 | 0.09 | 1.93 |
|  |  |  | Range | 17.74-23.82 | 28.70-39.31 | 47.37-60.49 | 16.57-20.87 | 8.11-64.99 | 0.96-1.26 | 16.85-22.35 |
| TD6 | 4 | M_2_ | Mean | 19.51 | 29.38 | 48.89 | 16.80 | 60.01 | 1.16 | 21.60 |
|  |  |  | SD | 3.15 | 4.74 | 5.54 | 0.60 | 5.56 | 0.17 | 4.06 |
|  |  |  | Range | 16.34-22.98 | 25.14-33.74 | 41.48-54.54 | 16.03-17.47 | 52.53-65.98 | 1.02-1.37 | 17.65-27.25 |
| HER | 4 |  | Mean | 23.18 | 33.65 | 56.83 | 18.23 | 59.03 | 1.27 | 22.15 |
|  |  |  | SD | 1.35 | 5.11 | 5.82 | 1.34 | 3.10 | 0.09 | 2.71 |
|  |  |  | Range | 21.20-24.10 | 30.60-41.30 | 52.40-65.40 | 17.40-20.20 | 56.04-63.15 | 1.19-1.38 | 18.56-24.93 |
| NAH_Tf | 1 |  |  | 25.2 | 47.7 | 72.9 | 21.1 | 65.43 | 1.19 | 17.29 |
| AT-3179 |  |  |  | 21.58 | 32.93 | 54.51 | 17.66 | 60.41 | 1.22 | 21.29 |
| AT-169 |  |  |  | 15.78 | 23.39 | 39.17 | 13.96 | 59.71 | 1.13 | 23.37 |
| AT-271 |  |  |  | 16.49 | 27.21 | 43.70 | 16.15 | 62.27 | 1.02 | 19.57 |
| AT-284 |  |  |  | 17.76 | 26.08 | 43.84 | 14.75 | 59.49 | 1.20 | 23.58 |
| AT-1761 |  |  |  | 18.56 | 26.14 | 44.70 | 15.66 | 58.48 | 1.19 | 23.18 |
| AT-941 |  |  |  | 22.14 | 30.16 | 52.30 | 16.73 | 57.67 | 1.32 | 24.10 |
| AT-946 |  |  |  | 22.00 | 36.51 | 58.51 | 17.71 | 62.40 | 1.24 | 20.56 |
| AT-2270 |  |  |  | 17.76 | 28.66 | 46.42 | 16.27 | 61.74 | 1.09 | 20.39 |
| AT-2396 |  |  |  | 20.48 | 24.98 | 45.46 | 14.48 | 54.95 | 1.41 | 28.30 |
| AT-6579 |  |  |  | 20.73 | 32.18 | 52.91 | 17.59 | 60.82 | 1.18 | 20.77 |
| **SH** | **10** |  | **Mean** | **19.33** | **28.82** | **48.15** | **16.10** | **59.79** | **1.20** | **22.51** |
|  |  |  | **SD** | **2.35** | **4.09** | **6.04** | **1.37** | **2.31** | **0.11** | **2.58** |
|  |  |  | **Range** | **21.20-24.10** | **23.39-36.51** | **39.17-58.51** | **13.96-17.71** | **54.95-62.40** | **1.02-1.41** | **19.57-28.30** |
| MPEH_M-LN | 1 |  |  | 22.55 | 38.67 | 61.22 | 19.37 | 63.16 | 1.16 | 18.72 |
| NEA | 9 |  | Mean | 20.52 | 42.03 | 62.55 | 20.30 | 67.25 | 1.01 | 15.59 |
|  |  |  | SD | 2.93 | 4.78 | 7.57 | 1.97 | 1.28 | 0.07 | 0.91 |
|  |  |  | Range | 21.20-24.10 | 33.85-47.56 | 50.95-70.90 | 17.78-22.89 | 65.28-69.69 | 0.90-1.19 | 14.21-22.24 |
| *MH | 45 |  | Mean | 34.33 | 22.05 | 56.38 | 18.52 | 60.78 | 1.19 | 20.51 |
|  |  |  | SD | 4.26 | 2.59 |  | 1.24 | 3.249 | 0.14 | 2.93 |
|  |  |  | Range | 23.75-42.24 | 16.81-29.13 |  | 15.22-21.60 | 53.21-67.80 | 0.94-1.55 | 14.85-27.66 |
| MH | 21 |  | Mean | 22.61 | 32.34 | 54.95 | 18.13 | 58.73 | 1.25 | 22.14 |
|  |  |  | SD | 3.03 | 5.14 | 7.06 | 1.39 | 3.56 | 0.15 | 3.11 |
|  |  |  | Range | 17.27-28.31 | 22.26-43.73 | 39.92-65.87 | 15.35-20.64 | 50.76-66.39 | 1.02-1.53 | 16.22-28.58 |
| TD6 | 3 | M_3_ | Mean | 16.85 | 24.36 | 41.21 | 14.71 | 58.11 | 1.14 | 24.00 |
|  |  |  | SD | 3.74 | 9.06 | 12.76 | 2.68 | 5.02 | 0.05 | 4.50 |
|  |  |  | Range | 21.20-24.10 | 13.97-30.65 | 26.69-50.65 | 11.66-16.68 | 52.34-61.47 | 1.09-1.20 | 21.16-29.19 |
| HER | 1 |  |  | 15.9 | 24.1 | 40 | 16.3 | 60.25 | 0.97 | 19.87 |
| AT-30 |  |  |  | 20.10 | 26.23 | 46.33 | 16.70 | 56.62 | 1.20 | 23.50 |
| AT-811 |  |  |  | 15.17 | 31.19 | 46.36 | 16.28 | 67.28 | 0.93 | 16.68 |
| AT-143 |  |  |  | 23.24 | 22.89 | 46.13 | 15.82 | 49.62 | 1.47 | 30.71 |
| AT-1468 |  |  |  | 21.22 | 24.45 | 45.67 | 15.53 | 53.54 | 1.37 | 27.63 |
| AT-599 |  |  |  | 19.29 | 20.41 | 39.70 | 13.35 | 51.41 | 1.44 | 31.98 |
| AT-942 |  |  |  | 20.66 | 28.86 | 49.52 | 15.64 | 58.28 | 1.32 | 24.59 |
| AT-1959 |  |  |  | 18.70 | 26.60 | 45.30 | 15.98 | 58.72 | 1.17 | 22.69 |
| AT-2438b |  |  |  | 19.90 | 22.09 | 41.99 | 13.47 | 52.61 | 1.48 | 31.43 |
| AT-2273 |  |  |  | 21.45 | 23.44 | 44.89 | 15.05 | 52.22 | 1.43 | 29.44 |
| AT-2777 |  |  |  | 22.31 | 26.96 | 49.27 | 16.08 | 54.72 | 1.39 | 26.72 |
| AT-3182 |  |  |  | 22.06 | 26.77 | 48.83 | 15.69 | 54.82 | 1.41 | 27.17 |
| AT-3943 |  |  |  | 20.56 | 17.94 | 38.50 | 16.15 | 46.60 | 1.27 | 30.06 |
| **SH** | **12** |  | **Mean** | **20.39** | **24.82** | **45.21** | **15.48** | **54.70** | **1.32** | **26.88** |
|  |  |  | **SD** | **2.09** | **3.70** | **3.54** | **1.05** | **5.27** | **0.16** | **4.44** |
|  |  |  | **Range** | **21.20-24.10** | **17.94-31.19** | **38.50-49.52** | **13.35-16.70** | **46.60-67.28** | **0.93-1.48** | **16.68-31.98** |
| MPEH | 1 |  |  |  |  |  |  |  | 1.27 | 21.6 |
| MPEH_M-LN | 1 |  |  | 20.23 | 33.09 | 53.32 | 17.33 | 62.05 | 1.16 | 20.29 |
| MPEH_BH | 1 |  |  | 15.22 | 27.48 | 42.70 | 16.26 | 64.36 | 0.94 | 17.86 |
| NEA | 11 |  | Mean | 19.19 | 35.47 | 54.66 | 18.62 | 64.73 | 1.03 | 17.43 |
|  |  |  | SD | 2.52 | 6.06 | 7.79 | 1.57 | 2.96 | 0.08 | 1.92 |
|  |  |  | Range | 21.20-24.10 |  |  |  |  |  |  |
| *MH | 44 |  | Mean | 33.09 | 22.58 |  | 18.27 | 59.31 | 1.24 | 21.63 |
|  |  |  | SD | 5.11 | 3.28 |  | 1.36 | 3.15 | 0.15 | 2.99 |
|  |  |  | Range | 24.40-45.98 | 16.75-29.42 |  | 15.90-22.26 | 50.82-64.61 | 0.98-1.67 | 17.22-31.84 |
| MH | 17 |  | Mean | 22.02 | 31.02 | 53.04 | 18.08 | 58.42 | 1.22 | 22.02 |
|  |  |  | SD | 2.04 | 3.36 | 4.43 | 1.05 | 2.87 | 0.12 | 2.68 |
|  |  |  | Range | 18.14-26.74 | 24.32-37.22 | 43.22-60.47 | 16.22-19.69 | 52.05-63.83 | 1.03-1.50 | 17.56-29.09 |

Upper molars. TD6: *H. antecessor* from Gran Dolina [41]. AT & SH: Sima de los Huesos (original data). HER: *H. erectus* [30, 44, 45, 50]. EMPH: European Middle Pleistocene *Homo* (St: Steinheim[30]). MPAH: Middle Pleistocene African *Homo* (TQ: Thomas Quarry [30]). NEA: Neanderthals [25, 28]. FHS: fossil *H. sapiens* (Qz: Qafzeh [30]). *MH: modern humans [47]. *Please note that in Smith et al., [47] the modern humans’ data does not include individual values, therefore we only employed it for comparative purposes but it was not possible to include it in the boxplots or statistical analyses. MH: modern humans [48 and original data].

Lower molars. TD6: *H. antecessor* from Gran Dolina [41]. EAH: East African *Homo* [53]. NAH: North African *Homo* (Tf: Tighenif [46]). HER: *H. erectus* [45]. AT & SH: Sima de los Huesos (original data). EMPH: European Middle Pleistocene *Homo* (MR: Mauer, [30]; M-LN: Mountmaurin [54]; BH: Mala Balanica, [56]). NEA: Neanderthals [25, 28]. *MH: modern humans [47]. *Please note that in Smith et al., [47] the modern humans’ data does not include individual values, therefore we only employed it for comparative purposes but it was not possible to include it in the boxplots or statistical analyses. MH: modern humans [49].
