# Supplementary material for: Crown tissue proportions and enamel thickness distribution in the Middle Pleistocene hominin molars from Sima de los Huesos (SH) population (Atapuerca, Spain)
Source: PLoS One. 2020 Jun 8;15(6):e0233281. doi: 10.1371/journal.pone.0233281 (PMC7279586; doi:10.1371/journal.pone.0233281)
Supplement: S2 Table — (DOCX) [file pone.0233281.s007.docx]

S2 Table. 3D enamel thickness, complete crown, values measured in the SH maxillary and mandibular molars and those of the extinct and extant specimens/populations.

| Sample | N | Tooth Class |  | Ve (mm3) | Vcdp (mm3) | Vc (mm3) | SEDJ (mm2) | Vcdp/Vc (%) | 3D AET (mm) | 3D RET |
| --- | --- | --- | --- | --- | --- | --- | --- | --- | --- | --- |
| TD6 | 2 | M^1^ | Mean | 301.75 | 345.67 | 647.41 | 233.45 | 53.11 | 1.30 | 18.69 |
|  |  |  | SD | 31.88 | 80.73 | 112.61 | 40.68 | 3.23 | 0.09 | 2.75 |
|  |  |  | Range | 279.20-324.29 | 288.402.75 | 567.79-727.04 | 204.69-262.22 | 50.83-55.40 | 1.24-1.36 | 16.75-20.64 |
| HER | 1 |  |  | 275.00 | 331.60 | 606.60 | 243.80 | 54.67 | 1.13 | 16.30 |
| AT-599 |  |  |  | 240.71 | 251.17 | 491.88 | 184.79 | 51.06 | 1.62 | 1.30 |
| AT-2071 |  |  |  | 257.76 | 264.36 | 522.12 | 197.77 | 50.63 | 1.17 | 1.30 |
| AT-3177 |  |  |  | **307.38** | 359.52 | 666.90 | 240.14 | 53.91 | 1.28 | 18.00 |
| **SH** | **3** |  | **Mean** | **268.62** | **291.68** | **560.30** | **207.57** | **51.87** | **1.30** | **19.65** |
|  |  |  | **SD** | **34.64** | **59.12** | **93.55** | **28.95** | **1.78** | **0.01** | **1.44** |
|  |  |  | **Range** | **240.71-307.38** | **251.17-359.52** | **491.88-669.90** | **180.79-240.14** | **50.63-53.91** | **1.28-1.30** | **18.00-20.65** |
| NEA | 4 |  | Mean | 275.14 | 345.56 | 620.70 | 243.96 | 55.36 | 1.14 | 16.39 |
|  |  |  | SD | 44.77 | 84.27 | 125.27 | 50.28 | 3.39 | 0.07 | 2.20 |
|  |  |  | Range | 249.44-341.92 | 272.83-460.43 | 532.31-802.35 | 208.03-317.81 | 51.25-58.77 | 1.07-1.20 | 13.93-18.41 |
| MH | 13 |  | Mean | 195.02 | 238.00 | 433.02 | 174.97 | 54.54 | 1.14 | 18.61 |
|  |  |  | SD | 25.26 | 51.87 | 68.75 | 33.41 | 4.68 | 0.20 | 3.67 |
|  |  |  | Range | 162.55-229.89 | 151.17-304.19 | 317.77-534.08 | 134.91-255.00 | 47.57-61.52 | 0.84-1.58 | 12.63-23.52 |
|  |  | M^2^ |  |  |  |  |  |  |  |  |
| TD6 | 2 |  | Mean | 299.98 | 311.99 | 611.97 | 209.67 | 50.92 | 1.44 | 21.30 |
|  |  |  | SD | 2.37 | 31.13 | 28.76 | 23.33 | 2.69 | 0.17 | 3.24 |
|  |  |  | Range | 298.30-301.65 | 289.98-334.00 | 591.63-632.30 | 193.17-226.17 | 48.24-54.14 | 1.32-1.54 | 19.01-23.59 |
| AT-12 |  |  |  | 191.81 | 208.07 | 399.88 | 168.427 | 52.03 | 1.14 | 19.22 |
| AT-824 |  |  |  | 226.48 | 230.97 | 457.45 | 180.27 | 50.49 | 1.26 | 20.48 |
| AT-817 |  |  |  | 188.9 | 212.59 | 401.49 | 160.24 | 52.95 | 1.18 | 19.75 |
| AT-15 |  |  |  | 288.07 | 311.85 | 599.92 | 215.17 | 51.98 | 1.34 | 19.74 |
| AT-170 |  |  |  | 217.51 | 228.55 | 446.06 | 176.7 | 51.24 | 1.23 | 20.13 |
| AT-960 |  |  |  | 232.07 | 216.30 | 448.37 | 162.83 | 48.24 | 1.43 | 23.74 |
| AT-822 |  |  |  | 222.81 | 263.09 | 485.90 | 188.71 | 54.14 | 1.18 | 18.43 |
| AT-2175 |  |  |  | 241.34 | 255.85 | 497.19 | 188.42 | 51.46 | 1.28 | 20.18 |
| AT-6215 |  |  |  | 182.41 | 190.12 | 372.53 | 161.1 | 51.03 | 1.13 | 19.69 |
| **SH** | **9** |  | **Mean** | **221.27** | **235.27** | **456.54** | **177.99** | **51.51** | **1.24** | **20.15** |
|  |  |  | **SD** | **32.50** | **36.71** | **67.59** | **17.80** | **1.64** | **0.10** | **1.47** |
|  |  |  | **Range** | **143.12-288.07** | **158.78-311.85** | **301.9-599.92** | **135.97-215.17** | **48.24-54.14** | **1.05-1.43** | **18.43-23.74** |
| NEA | 6 |  | Mean | 242.55 | 342.92 | 585.47 | 223.06 | 58.18 | 1.10 | 15.91 |
|  |  |  | SD | 18.41 | 67.57 | 77.22 | 28.49 | 4.48 | 0.13 | 2.94 |
|  |  |  | Range | 210.06-256.45 | 260.83-418.51 | 480.29-669.50 | 185.00-250.31 | 50.70-62.80 | 0.97-1.33 | 13.24-20.88 |
| MH | 14 |  | Mean | 217.52 | 227.01 | 444.53 | 165.98 | 51.03 | 1.33 | 21.93 |
|  |  |  | SD | 32.03 | 35.28 | 59.90 | 26.19 | 3.49 | 0.19 | 3.94 |
|  |  |  | Range | 179.57-293.86 | 179.58-284.08 | 368.13-577.94 | 105.67-218.18 | 43.24-58.46 | 0.96-1.78 | 15.06-31.63 |
|  |  | M^3^ |  |  |  |  |  |  |  |  |
| HER | 1 |  |  | 195.50 | 144.20 | 339.70 | 134.90 | 42.45 | 1.45 | 27.64 |
| AT-10 |  |  |  | 170.62 | 176.36 | 346.98 | 143.19 | 50.83 | 1.19 | 21.25 |
| AT-194 |  |  |  | 202.433 | 173.61 | 376.04 | 144.60 | 46.17 | 1.40 | 25.10 |
| AT-601 |  |  |  | 185.74 | 165.63 | 351.37 | 138.64 | 47.14 | 1.34 | 24.40 |
| AT-805 |  |  |  | 213.77 | 185.95 | 399.72 | 147.00 | 46.52 | 1.45 | 25.48 |
| AT-826 |  |  |  | 134.63 | 120.8 | 255.43 | 113.16 | 47.29 | 1.19 | 24.07 |
| AT-3181 |  |  |  | 219.63 | 180.6 | 400.23 | 146.01 | 45.12 | 1.50 | 26.61 |
| AT-1471 |  |  |  | 143.67 | 120.48 | 264.15 | 112.32 | 45.61 | 1.28 | 25.90 |
| AT-2393 |  |  |  | 173.50 | 157.37 | 330.87 | 138.50 | 47.56 | 1.25 | 23.20 |
| AT-3183 |  |  |  | 155.9 | 128.8 | 284.70 | 114.96 | 45.24 | 1.36 | 26.85 |
| AT-5082 |  |  |  | 220.35 | 173.74 | 394.09 | 146.23 | 44.09 | 1.51 | 27.00 |
| AT-5292 |  |  |  | 185.88 | 156.94 | 342.82 | 130.69 | 45.78 | 1.42 | 26.37 |
| AT-274 |  |  |  | 168.31 | 119.06 | 287.37 | 113.93 | 41.43 | 1.48 | 30.03 |
| AT-602 |  |  |  | 198.63 | 176.93 | 375.56 | 144.30 | 47.11 | 1.38 | 24.52 |
| AT-6215 |  |  |  | 170.19 | 142.18 | 312.37 | 132.12 | 45.52 | 1.29 | 24.68 |
| **SH** | **14** |  | **Mean** | **181.66** | **155.60** | **337.26** | **133.26** | **46.10** | **1.36** | **25.39** |
|  |  |  | **SD** | **27.09** | **24.60** | **49.88** | **13.82** | **2.08** | **0.11** | **2.06** |
|  |  |  | **Range** | **134.63-220.35** | **119.06-185.95** | **255.43-400.23** | **112.32-147.00** | **41.43-50.82** | **1.18-1.50** | **21.24-30.02** |
| NEA | 9 |  | Mean | 207.85 | 294.26 | 502.11 | 201.06 | 58.47 | 1.03 | 15.62 |
|  |  |  | SD | 39.23 | 68.30 | 100.36 | 28.11 | 3.54 | 0.14 | 2.05 |
|  |  |  | Range | 115.51-259.00 | 186.76-421.54 | 302.27-637.61 | 153.57-248.17 | 54.06-66.11 | 0.75-1.18 | 11.61-18.43 |
| MH | 22 |  | Mean | 201.77 | 176.80 | 378.57 | 143.74 | 46.44 | 1.44 | 26.10 |
|  |  |  | SD | 37.78 | 42.68 | 74.92 | 37.23 | 4.35 | 0.24 | 5.14 |
|  |  |  | Range | 139.67-317.72 | 103.53-288.09 | 273.35-605.81 | 82.48-246.78 | 37.83-52.98 | 0.91-1.94 | 14.54-34.10 |
|  |  | M_1_ |  |  |  |  |  |  |  |  |
| TD6 | 3 |  | Mean | 260.27 | 341.04 | 601.31 | 229.20 | 56.89 | 1.13 | 16.16 |
|  |  |  | SD | 58.41 | 62.50 | 117.37 | 31.48 | 2.80 | 0.13 | 1.42 |
|  |  |  | Range | 193.92-303.92 | 284.32-408.04 | 478.24-712.01 | 197.01-259.92 | 53.90-59.45 | 0.98-1.23 | 14.97-17.74 |
| EAH_Tf | 1 |  |  | 261.40 | 332.20 | 593.60 | 256.10 | 55.96 | 1.02 | 14.74 |
| NAH_MA93 | 1 |  |  | 278.19 | 469.32 | 747.51 | 298.16 | 62.78 | 0.93 | 12.01 |
| **AT-829** | **1** |  |  | **219.75** | **260.35** | **480.10** | **197.53** | **54.23** | **1.11** | **17.74** |
| NEA | 12 |  | Mean | 245.48 | 346.93 | 592.41 | 223.31 | 58.47 | 1.13 | 16.29 |
|  |  |  | SD | 39.73 | 61.55 | 91.94 | 48.81 | 3.53 | 0.23 | 3.72 |
|  |  |  | Range | 189.76-309.52 | 264.35-447.75 | 490.05-739.37 | 129.33-302.55 | 52.01-63.52 | 0.82-1.63 | 11.79-24.02 |
| LV | 1 |  |  | 270.51 | 292.49 | 563 | 202.35 | 51.95 | 1.34 | 20.14 |
| MH | 19 |  | Mean | 232.90 | 262.03 | 494.94 | 206.59 | 52.64 | 1.14 | 17.97 |
|  |  |  | SD | 40.34 | 62.23 | 99.66 | 35.34 | 3.10 | 0.13 | 2.65 |
|  |  |  | Range | 168.41-349.88 | 172.59-420.84 | 372.18-770.73 | 154.94-271.13 | 46.37-57.35 | 0.92-1.36 | 14.46-22.21 |
|  |  |  |  |  |  |  |  |  |  |  |
|  |  | M_2_ |  |  |  |  |  |  |  |  |
| TD6 | 4 |  | Mean | 256.54 | 288.73 | 545.27 | 204.07 | 51.70 | 1.27 | 19.85 |
|  |  |  | SD | 39.39 | 100.75 | 139.57 | 40.09 | 6.60 | 0.09 | 4.26 |
|  |  |  | Range | 200.92-292.18 | 146.35-360.80 | 347.27-652.98 | 146.44-234.89 | 42.14-56.80 | 1.16-1.37 | 16.36-26.04 |
| HER | 4 |  | Mean | 261.80 | 246.95 | 508.75 | 196.30 | 48.40 | 1.34 | 21.42 |
|  |  |  | SD | 16.20 | 34.88 | 49.68 | 19.11 | 2.24 | 0.07 | 1.94 |
|  |  |  | Range | 240.80-279.70 | 219.20-291.90 | 460.00-571.60 | 181.10-222.20 | 45.77-51.07 | 1.26-1.42 | 18.98-23.60 |
| NAH | 1 |  |  | 373.06 | 502.42 | 875.48 | 312.54 | 57.39 | 1.19 | 15.01 |
| AT-3179 |  |  |  | 234.04 | 254.79 | 488.83 | 183.52 | 52.12 | 1.28 | 20.12 |
| AT-169 |  |  |  | 179.56 | 176.96 | 356.52 | 141.71 | 49.64 | 1.27 | 22.57 |
| AT-271 |  |  |  | 181.49 | 170.27 | 351.76 | 147.19 | 48.41 | 1.23 | 22.25 |
| AT-284 |  |  |  | 192.86 | 176.84 | 369.70 | 144.35 | 47.83 | 1.34 | 23.80 |
| AT-1761 |  |  |  | 183.37 | 166.73 | 350.1 | 137.63 | 47.62 | 1.33 | 24.21 |
| AT-941 |  |  |  | 248.33 | 254.62 | 502.95 | 184.45 | 50.63 | 1.35 | 21.24 |
| AT-946 |  |  |  | 273.38 | 282.26 | 555.64 | 198.95 | 50.80 | 1.37 | 20.95 |
| AT-2270 |  |  |  | 174.76 | 208.47 | 383.23 | 157.29 | 54.40 | 1.11 | 18.74 |
| AT-2396 |  |  |  | 203.14 | 166.35 | 369.49 | 135.53 | 45.02 | 1.50 | 27.25 |
| AT-6579 |  |  |  | 224.64 | 226.74 | 451.38 | 176.31 | 50.23 | 1.27 | 20.89 |
| **SH** | **10** |  | **Mean** | **209.56** | **208.40** | **417.96** | **160.69** | **49.67** | **1.30** | **22.20** |
|  |  |  | **SD** | **33.84** | **43.49** | **75.25** | **23.04** | **2.62** | **0.10** | **2.42** |
|  |  |  | **Range** | **174.76-273.38** | **166.35-282.26** | **350.10-555.64** | **135.53-198.95** | **45.02-54.40** | **1.11-1.50** | **18.74-27.25** |
| MPEH_M-LN | 1 |  |  | 244.8 | 251.03 | 495.83 | 179.8 | 50.63 | 1.36 | 21.58 |
| NEA | 11 |  | Mean | 240.57 | 356.44 | 597.01 | 226.77 | 59.50 | 1.06 | 15.17 |
|  |  |  | SD | 56.24 | 96.05 | 142.48 | 43.23 | 4.63 | 0.16 | 2.69 |
|  |  |  | Range | 169.13-308.44 | 250.03-519.34 | 436.10-808.61 | 182.48-302.91 | 50.97-67.53 | 0.81-1.32 | 11.88-20.92 |
| MH | 35 |  | Mean | 246.43 | 258.03 | 509.57 | 196.79 | 50.51 | 1.29 | 20.55 |
|  |  |  | SD | 44.63 | 59.77 | 102.05 | 42.99 | 4.45 | 0.29 | 5.13 |
|  |  |  | Range | 161.65-361.91 | 135.34-426.01 | 317.28-770.04 | 95.40-285.35 | 36.56-57.32 | 0.65-2.30 | 12.56-40.71 |
|  |  | M_3_ |  |  |  |  |  |  |  |  |
| TD6 | 3 |  | Mean | 182.95 | 167.70 | 350.65 | 143.96 | 44.63 | 1.29 | 25.74 |
|  |  |  | SD | 64.41 | 104.37 | 167.76 | 56.88 | 10.02 | 0.08 | 8.62 |
|  |  |  | Range | 109.39-229.2 | 55.52-261.93 | 164.91-491.15 | 81.15-191.97 | 33.67-53.33 | 1.19-1.35 | 18.66-35.33 |
| HER | 1 |  |  | 174.8 | 195.50 | 370.30 | 167.2 | 52.80 | 1.05 | 18.01 |
| NAH | 2 |  | Mean | 280.52 | 359.04 | 639.55 | 250.99 | 57.34 | 1.11 | 15.59 |
|  |  |  | SD | 129.72 | 17.67 | 147.39 | 6.35 | 10.45 | 0.49 | 6.62 |
|  |  |  | Range | 188.79-372.24 | 346.54-371.53 | 535.33-743.77 | 246.5-255.48 | 49.95-64.73 | 0.77-1.46 | 10.90-20.27 |
| AT-30 |  |  |  | 196.72 | 181.82 | 378.54 | 148.03 | 48.03 | 1.33 | 23.46 |
| AT-811 |  |  |  | 169.39 | 229.18 | 398.57 | 161.69 | 57.50 | 1.05 | 17.12 |
| AT-143 |  |  |  | 221.17 | 191.13 | 412.30 | 148.9 | 46.36 | 1.49 | 25.79 |
| AT-1468 |  |  |  | 206.78 | 185.12 | 391.90 | 143.8 | 47.24 | 1.44 | 25.23 |
| AT-599 |  |  |  | 192.31 | 159.05 | 351.36 | 128.61 | 45.27 | 1.50 | 27.60 |
| AT-942 |  |  |  | 243.94 | 223.23 | 467.17 | 168.73 | 47.78 | 1.45 | 23.83 |
| AT-1959 |  |  |  | 195.74 | 195.82 | 391.56 | 147.7 | 50.01 | 1.33 | 22.82 |
| AT-2438b |  |  |  | 194.04 | 157.57 | 351.61 | 128.78 | 44.81 | 1.51 | 27.90 |
| AT-2273 |  |  |  | 213.32 | 175.75 | 389.07 | 143.00 | 45.17 | 1.49 | 26.63 |
| AT-2777 |  |  |  | 237.19 | 212.61 | 449.80 | 155.45 | 47.27 | 1.53 | 25.56 |
| AT-3182 |  |  |  | 221.38 | 203.19 | 424.57 | 149.63 | 47.86 | 1.48 | 25.17 |
| AT-3943 |  |  |  | 175.64 | 162.49 | 338.13 | 137.37 | 48.06 | 1.28 | 23.43 |
| **SH** | **12** |  | **Mean** | **205.64** | **189.75** | **395.38** | **146.81** | **47.95** | **1.40** | **24.54** |
|  |  |  | **SD** | **22.79** | **24.16** | **39.02** | **11.91** | **3.35** | **0.14** | **2.85** |
|  |  |  | **Range** | **169.39-243.94** | **157.57-229.18** | **338.13-467.17** | **128.61-168.73** | **44.81-57.50** | **1.05-1.53** | **17.12-27.90** |
| MPEH_M-LN | 1 |  |  | 264.01 | 246.84 | 510.85 | 181.19 | 48.32 | 1.46 | 23.23 |
| NEA | 11 |  | Mean | 211.65 | 293.39 | 505.04 | 187.16 | 57.77 | 1.14 | 17.31 |
|  |  |  | SD | 34.66 | 70.87 | 81.51 | 21.09 | 6.08 | 0.19 | 3.30 |
|  |  |  | Range | 154.04-262.18 | 228.03-485.90 | 407.80-708.01 | 142.85-228.03 | 49.11-68.63 | 0.82-1.41 | 12.74-22.28 |
| MH | 20 |  | Mean | 232.09 | 223.86 | 455.95 | 171.91 | 48.53 | 1.36 | 22.82 |
|  |  |  | SD | 53.13 | 74.10 | 124.08 | 38.84 | 3.87 | 0.18 | 3.53 |
|  |  |  | Range | 166.58-362.14 | 145.16-405.48 | 328.23-767.63 | 121.09-273.48 | 42.18-55.44 | 1.08-1.85 | 17.78-30.20 |

Upper molars: TD6: *H. antecessor* from Gran Dolina [41]. HER: *H. erectus* [45]. AT & SH: Sima de los Huesos (original data). NEA: Neanderthals [25, 28, 51]. MH: modern humans [25, 41 and original data].

Lower molars: TD6: *H. antecessor* from Gran Dolina [41]. NAH: North African *Homo* (Tf: Tighenif [46]). EAH: East African *Homo* (MA93: Buia [53]). AT & SH: Sima de los Huesos (original data). HER: *H. erectus* [45]. NEA: Neanderthals [25, 28]. LV: Lagar Velho (Original data from Nespos). MH: modern humans [25, 41, 55 and original data].
