# Supplementary material for: Crown tissue proportions and enamel thickness distribution in the Middle Pleistocene hominin molars from Sima de los Huesos (SH) population (Atapuerca, Spain)
Source: PLoS One. 2020 Jun 8;15(6):e0233281. doi: 10.1371/journal.pone.0233281 (PMC7279586; doi:10.1371/journal.pone.0233281)
Supplement: S3 Table — (DOCX) [file pone.0233281.s008.docx]

S3 Table. 3D lateral enamel thickness values measured in the SH maxillary and mandibular molars and those of the extinct and extant specimens/populations.

| Sample | N | Tooth class |  | VLe (mm3) | VLcdp (mm3) | VLc (mm3) | LSEDJ (mm2) | VLcdp/Vlc (%) | 3D LAET (mm) | 3D LRET |
| --- | --- | --- | --- | --- | --- | --- | --- | --- | --- | --- |
| TD6 | 4 | M^1^ | Mean | 54.60 | 248.11 | 302.70 | 97.49 | 81.57 | 0.57 | 9.37 |
|  |  |  | SD | 11.85 | 77.92 | 89.36 | 28.44 | 1.88 | 0.06 | 2.12 |
|  |  |  | Range | 37.48-64.47 | 144.82-333.63 | 182.30-398.10 | 58.12-125.71 | 79.44-83.81 | 0.51-0.64 | 7.93-12.28 |
| HER | 1 |  |  | 44.20 | 186.33 | 230.53 | 85.56 | 80.83 | 0.52 | 9.05 |
| NAH | 1 |  |  | 102.82 | 389.11 | 491.93 | 151.14 | 79.10 | 0.68 | 9.32 |
| MPEH | 1 |  |  | 60.77 | 239.61 | 300.37 | 110.08 | 79.77 | 0.55 | 8.89 |
| AT-20 |  |  |  | 66.2 | 240.16 | 306.36 | 93.43 | 78.39 | 0.71 | 11.40 |
| AT-26 |  |  |  | 68.05 | 208.62 | 276.67 | 89.43 | 75.40 | 0.76 | 12.83 |
| AT-196 |  |  |  | 53.28 | 194.38 | 247.66 | 83.17 | 78.49 | 0.64 | 11.06 |
| AT-812 |  |  |  | 46.4 | 138.86 | 185.26 | 68.08 | 74.95 | 0.68 | 13.16 |
| AT-959 |  |  |  | 74.81 | 197.98 | 272.79 | 87.33 | 72.58 | 0.86 | 14.70 |
| AT-2071 |  |  |  | 64.74 | 198.72 | 263.46 | 82.34 | 75.43 | 0.79 | 13.47 |
| AT-3177 |  |  |  | 75.72 | 264.31 | 340.03 | 101.52 | 77.73 | 0.75 | 11.62 |
| AT-5804 |  |  |  | 53.31 | 209.34 | 262.65 | 86.04 | 79.70 | 0.62 | 10.43 |
| **SH** | **8** |  | **Mean** | **54.84** | **187.33** | **242.17** | **82.89** | **77.33** | **0.66** | **11.56** |
|  |  |  | **SD** | **12.16** | **42.16** | **54.01** | **13.84** | **1.07** | **0.04** | **0.63** |
|  |  |  | **Range** | **40.49-78.15** | **148.94-278.59** | **189.43-356.74** | **68.17-109.30** | **75.11-78.63** | **0.59-0.72** | **10.95-13.07** |
| MPEH_M-LN | 1 |  |  | 20.63 | 77.34 | 97.97 | 31.98 | 78.94 | 0.65 | 15.14 |
| NEA | 10 |  | Mean | 74.45 | 294.03 | 368.49 | 126.56 | 80.15 | 0.58 | 8.67 |
|  |  |  | SD | 24.82 | 67.89 | 90.76 | 23.49 | 2.78 | 0.11 | 1.30 |
|  |  |  | Range | 36.07-108.44 | 205.37-404.52 | 241.45-508.55 | 92.80-159.88 | 73.84-85.06 | 0.39-0.79 | 6.59-11.65 |
| LV | 1 |  |  | 54.09 | 234.55 | 288.64 | 96.99 | 81.26 | 0.56 | 9.04 |
| MH | 21 |  | Mean | 49.11 | 184.84 | 234.02 | 87.92 | 79.13 | 0.54 | 9.70 |
|  |  |  | SD | 20.08 | 63.01 | 82.21 | 24.07 | 2.03 | 0.08 | 1.09 |
|  |  |  | Range | 14.91-113.81 | 52.61-362.55 | 67.51-476.37 | 38.17-156.12 | 75.81-83.00 | 0.39-0.73 | 7.54-11.68 |
|  |  | M^2^ |  |  |  |  |  |  |  |  |
| TD6 | 3 |  | Mean | 58.77 | 239.55 | 298.33 | 91.22 | 80.32 | 0.65 | 10.42 |
|  |  |  | SD | 9.18 | 29.95 | 37.30 | 12.30 | 1.76 | 0.08 | 1.38 |
|  |  |  | Range | 48.47-66.08 | 219.91-274.03 | 268.38-340.11 | 83.65-105.42 | 78.44-81.94 | 0.58-0.73 | 9.60-12.01 |
| EMPH_Vg | 1 |  |  | 76.34 | 242.16 | 318.50 | 134.09 | 76.03 | 0.57 | 9.13 |
| AT-12 |  |  |  | 29.68 | 145.36 | 175.04 | 63.23 | 83.04 | 0.47 | 8.93 |
| AT-824 |  |  |  | 36.8 | 153.54 | 190.34 | 65.2 | 80.67 | 0.56 | 10.54 |
| AT-817 |  |  |  | 41.31 | 162.43 | 203.74 | 73.68 | 79.72 | 0.56 | 10.28 |
| AT-15 |  |  |  | 59.79 | 224.85 | 284.64 | 90.56 | 78.99 | 0.66 | 10.86 |
| AT-170 |  |  |  | 47.2 | 165.25 | 212.45 | 74.24 | 77.78 | 0.64 | 11.59 |
| AT-960 |  |  |  | 54.98 | 168.53 | 223.51 | 77.11 | 75.40 | 0.71 | 12.91 |
| AT-822 |  |  |  | 50.95 | 198.09 | 249.04 | 85.12 | 79.54 | 0.60 | 10.27 |
| AT-2175 |  |  |  | 33.43 | 174.77 | 208.20 | 68.79 | 83.94 | 0.49 | 8.69 |
| AT-815 |  |  |  | 24.39 | 112.15 | 136.54 | 55.38 | 82.14 | 0.44 | 9.13 |
| AT-588 |  |  |  | 34.46 | 162.00 | 196.46 | 70.29 | 82.46 | 0.49 | 8.99 |
| AT-4336 |  |  |  | 61.03 | 232.71 | 293.74 | 93.63 | 79.22 | 0.65 | 10.60 |
| AT-6215 |  |  |  | 26.68 | 127.49 | 154.17 | 57.88 | 82.69 | 0.46 | 9.16 |
| **SH** | **12** |  | **Mean** | **41.73** | **168.93** | **210.66** | **72.93** | **80.47** | **0.56** | **10.16** |
|  |  |  | **SD** | **12.82** | **35.57** | **47.33** | **12.14** | **2.49** | **0.09** | **1.26** |
|  |  |  | **Range** | **24.39-61.03** | **112.15-232.71** | **136.54-293.74** | **55.38-93.63** | **75.40-83.94** | **0.44-0.71** | **8.69-12.91** |
| NEA | 7 |  | Mean | 69.45 | 312.06 | 381.51 | 112.15 | 79.39 | 0.61 | 9.38 |
|  |  |  | SD | 16.20 | 170.34 | 173.08 | 16.44 | 5.57 | 0.06 | 1.36 |
|  |  |  | Range | 48.86-94.32 | 199.678-678.03 | 251.18-741.58 | 89.75-140.79 | 74.60-91.43 | 0.54-0.70 | 6.63-11.03 |
| MH | 14 |  | Mean | 40.40 | 161.32 | 201.72 | 80.67 | 79.97 | 0.50 | 9.18 |
|  |  |  | SD | 9.71 | 34.93 | 43.04 | 12.73 | 2.22 | 0.06 | 1.08 |
|  |  |  | Range | 24.50-56.03 | 95.62-211.61 | 120.12-263.92 | 60.44-99.98 | 76.24-84.20 | 0.39-0.59 | 7.11-10.82 |
|  |  | M^3^ |  |  |  |  |  |  |  |  |
| HER | 3 |  | Mean | 57.45 | 178.47 | 235.93 | 82.32 | 76.20 | 0.67 | 12.03 |
|  |  |  | SD | 30.90 | 76.03 | 106.88 | 29.53 | 1.90 | 0.11 | 0.39 |
|  |  |  | Range | 35.35-92.76 | 119.27-264.22 | 154.62-356.98 | 60.51-115.92 | 74.01-77.45 | 0.58-0.80 | 11.75-12.47 |
| AT-10 |  |  |  | 38.08 | 138.04 | 176.12 | 69.81 | 78.38 | 0.55 | 10.55 |
| AT-194 |  |  |  | 40.31 | 127.82 | 168.13 | 63.15 | 76.02 | 0.64 | 12.67 |
| AT-601 |  |  |  | 25.76 | 113.38 | 139.14 | 55.94 | 81.49 | 0.46 | 9.51 |
| AT-805 |  |  |  | 44.03 | 141.27 | 185.30 | 68.93 | 76.24 | 0.64 | 12.26 |
| AT-826 |  |  |  | 23.04 | 85.46 | 108.50 | 47.36 | 78.76 | 0.49 | 11.04 |
| AT-819 |  |  |  | 37.69 | 154.04 | 191.73 | 69.86 | 80.34 | 0.54 | 10.06 |
| AT-3181 |  |  |  | 50.23 | 142.88 | 193.11 | 73.14 | 73.99 | 0.69 | 13.14 |
| AT-1471 |  |  |  | 28.73 | 93.74 | 122.47 | 54.24 | 76.54 | 0.53 | 11.66 |
| AT-2393 |  |  |  | 24.16 | 108.95 | 133.11 | 53.71 | 81.85 | 0.45 | 9.42 |
| AT-3183 |  |  |  | 31.36 | 97.50 | 128.86 | 54.21 | 75.66 | 0.58 | 12.57 |
| AT-5082 |  |  |  | 35.78 | 122.00 | 157.78 | 60.26 | 77.32 | 0.59 | 11.97 |
| AT-5292 |  |  |  | 45.28 | 121.86 | 167.14 | 66.56 | 72.91 | 0.68 | 13.72 |
| AT-274 |  |  |  | 30.53 | 85.53 | 116.06 | 48.52 | 73.69 | 0.63 | 14.28 |
| AT-602 |  |  |  | 29.88 | 123.92 | 153.80 | 59.91 | 80.57 | 0.50 | 10.00 |
| AT-6215 |  |  |  | 23.40 | 93.05 | 116.45 | 47.35 | 79.91 | 0.49 | 10.91 |
| **SH** | **15** |  | **Mean** | **33.88** | **116.63** | **150.51** | **59.53** | **77.58** | **0.56** | **11.59** |
|  |  |  | **SD** | **8.56** | **22.11** | **29.07** | **8.74** | **2.88** | **0.08** | **1.53** |
|  |  |  | **Range** | **23.04-50.23** | **85.46-154.04** | **108.50-193.11** | **47.35-73.14** | **72.91-81.85** | **0.45-0.69** | **9.42-14.28** |
| NEA | 5 |  | Mean | 56.76 | 217.81 | 274.57 | 97.22 | 79.59 | 0.58 | 9.60 |
|  |  |  | SD | 16.70 | 47.84 | 61.59 | 15.60 | 3.80 | 0.14 | 2.14 |
|  |  |  | Range | 27.44-69.00 | 163.62-292.61 | 191.05-361.62 | 79.12-121.81 | 76.37-85.64 | 0.35-0.68 | 6.34-11.37 |
| MH | 12 |  | Mean | 40.42 | 142.25 | 182.67 | 70.92 | 78.10 | 0.56 | 10.73 |
|  |  |  | SD | 15.33 | 36.57 | 50.82 | 12.21 | 2.48 | 0.10 | 1.31 |
|  |  |  | Range | 27.23-86.83 | 98.08-241.00 | 125.31-327.83 | 55.18-104.97 | 73.51-83.77 | 0.41-0.83 | 7.65-13.29 |
|  |  | M_1_ |  |  |  |  |  |  |  |  |
| TD6 | 4 |  | Mean | 54.60 | 248.11 | 302.70 | 97.49 | 81.57 | 0.57 | 9.37 |
|  |  |  | SD | 11.85 | 77.92 | 89.36 | 28.44 | 1.88 | 0.06 | 2.12 |
|  |  |  | Range | 37.48-64.47 | 144.82-333.63 | 182.30-398.10 | 58.12-125.71 | 79.44-83.81 | 0.51-0.64 | 7.93-12.28 |
| HER | 1 |  |  | 44.20 | 186.33 | 230.53 | 85.56 | 80.83 | 0.52 | 9.05 |
| NAH_Tig | 1 |  |  | 102.82 | 389.11 | 491.93 | 151.14 | 79.10 | 0.68 | 9.32 |
| MPEH_FR | 1 |  |  | 60.77 | 239.61 | 300.37 | 110.08 | 79.77 | 0.55 | 8.89 |
| AT-2 |  |  |  | 73.81 | 238.50 | 312.31 | 103.99 | 76.37 | 0.71 | 11.45 |
| AT-3933 |  |  |  | 58.99 | 211.30 | 270.29 | 90.18 | 78.18 | 0.65 | 10.98 |
| AT-101 |  |  |  | 47.03 | 171.32 | 218.35 | 76.38 | 78.46 | 0.62 | 11.09 |
| AT-141 |  |  |  | 46.24 | 161.29 | 207.53 | 74.46 | 77.72 | 0.62 | 11.41 |
| AT-272 |  |  |  | 46.84 | 155.03 | 201.87 | 73.37 | 76.80 | 0.64 | 11.88 |
| AT-829 |  |  |  | 56.94 | 202.49 | 259.43 | 88.00 | 78.05 | 0.65 | 11.02 |
| AT-1759 |  |  |  | 43.51 | 155.72 | 199.23 | 71.49 | 78.16 | 0.61 | 11.31 |
| AT-2276 |  |  |  | 54.04 | 176.84 | 230.88 | 78.40 | 76.59 | 0.69 | 12.28 |
| AT-2438 |  |  |  | 50.01 | 150.95 | 200.96 | 71.88 | 75.11 | 0.70 | 13.07 |
| AT-4318 |  |  |  | 70.00 | 234.87 | 304.87 | 99.48 | 77.04 | 0.70 | 11.40 |
| AT-21 |  |  |  | 78.15 | 278.59 | 356.74 | 109.30 | 78.09 | 0.72 | 10.95 |
| AT-576 |  |  |  | 46.90 | 149.44 | 196.34 | 72.48 | 76.11 | 0.65 | 12.19 |
| AT-561 |  |  |  | 40.49 | 148.94 | 189.43 | 68.17 | 78.63 | 0.59 | 11.21 |
| **SH** | **13** |  | **Mean** | **54.84** | **187.33** | **242.17** | **82.89** | **77.33** | **0.66** | **11.56** |
|  |  |  | **SD** | **12.16** | **42.16** | **54.01** | **13.84** | **1.07** | **0.04** | **0.63** |
|  |  |  | **Range** | **40.49-78.15** | **148.94-278.59** | **189.43-356.74** | **68.17-109.30** | **75.11-78.63** | **0.59-0.72** | **10.95-13.07** |
| MPEH_M-LN | 1 |  |  | 20.63 | 77.34 | 97.97 | 31.98 | 78.94 | 0.65 | 15.14 |
| NEA | 10 |  | Mean | 74.45 | 294.03 | 368.49 | 126.56 | 80.15 | 0.58 | 8.67 |
|  |  |  | SD | 24.82 | 67.89 | 90.76 | 23.49 | 2.78 | 0.11 | 1.30 |
|  |  |  | Range | 36.07-108.44 | 205.37-404.52 | 241.45-508.55 | 92.80-159.88 | 73.84-85.06 | 0.39-0.79 | 6.59-11.65 |
| LV | 1 |  | 1 | 54.09 | 234.55 | 288.64 | 96.99 | 81.26 | 0.56 | 9.04 |
| MH | 21 |  | Mean | 49.11 | 184.84 | 234.02 | 87.92 | 79.13 | 0.54 | 9.70 |
|  |  |  | SD | 20.08 | 63.01 | 82.21 | 24.07 | 2.03 | 0.08 | 1.09 |
|  |  |  | Range | 14.91-113.81 | 52.61-362.55 | 67.51-476.37 | 38.17-156.12 | 75.81-83.00 | 0.39-0.73 | 7.54-11.68 |
|  |  | M_2_ |  |  |  |  |  |  |  |  |
| TD6 | 4 |  | Mean | 46.29 | 219.11 | 265.4 | 82.77 | 82.05 | 0.57 | 9.99 |
|  |  |  | SD | 16.59 | 93.11 | 109.38 | 32.93 | 1.8 | 0.04 | 2.69 |
|  |  |  | Range | 22.26-58.74 | 88.12-297.87 | 110.353.56 | 35.98-107.78 | 79.83-84.25 | 0.54-0.62 | 8.04-13.90 |
| HER | 3 |  | Mean | 48.43 | 210.43 | 258.87 | 90.71 | 81.16 | 0.53 | 9.03 |
|  |  |  | SD | 5.03 | 37.16 | 42.03 | 10.72 | 1.25 | 0.02 | 0.65 |
|  |  |  | Range | 42.66-51.89 | 170.73-244.38 | 213.39-296.27 | 78.85-99.70 | 80.01-82.48 | 0.51-0.55 | 8.48-9.75 |
| AT-3179 |  |  |  | 60.97 | 204.87 | 265.84 | 90.20 | 77.07 | 0.68 | 11.47 |
| AT-169 |  |  |  | 47.38 | 148.65 | 196.03 | 74.74 | 75.83 | 0.63 | 11.97 |
| AT-271 |  |  |  | 41.66 | 123.98 | 165.64 | 68.35 | 74.85 | 0.61 | 12.22 |
| AT-284 |  |  |  | 41.28 | 139.14 | 180.42 | 66.28 | 77.12 | 0.62 | 12.02 |
| AT-1761 |  |  |  | 41.01 | 132.79 | 173.80 | 66.30 | 76.40 | 0.62 | 12.12 |
| AT-941 |  |  |  | 45.43 | 190.02 | 235.45 | 77.96 | 80.71 | 0.58 | 10.14 |
| AT-946 |  |  |  | 60.01 | 219.68 | 279.69 | 92.00 | 78.54 | 0.65 | 10.81 |
| AT-2270 |  |  |  | 31.73 | 152.16 | 183.89 | 64.44 | 82.75 | 0.49 | 9.22 |
| AT-2396 |  |  |  | 40.29 | 124.32 | 164.61 | 59.99 | 75.52 | 0.67 | 13.46 |
| AT-3176 |  |  |  | 69.01 | 244.45 | 313.46 | 102.87 | 77.98 | 0.67 | 10.73 |
| AT-3176 |  |  |  | 72.80 | 244.45 | 317.25 | 102.87 | 77.05 | 0.71 | 11.32 |
| AT-6579 |  |  |  | 60.62 | 185.68 | 246.30 | 86.17 | 75.39 | 0.70 | 12.33 |
| **SH** | **12** |  | **Mean** | **51.02** | **175.85** | **226.87** | **79.35** | **77.43** | **0.64** | **11.48** |
|  |  |  | **SD** | **13.09** | **45.05** | **57.07** | **15.11** | **2.32** | **0.06** | **1.13** |
|  |  |  | **Range** | **31.73-72.80** | **123.98-244.45** | **164.61-317.25** | **59.99-102.87** | **74.85-82.75** | **0.49-0.71** | **9.22-13.46** |
| MPEH_M-LN | 1 |  | 2 | 64.22 | 195.74 | 259.96 | 82.39 | 75.3 | 0.78 | 13.42 |
| NEA | 8 |  | Mean | 74.14 | 320.04 | 394.18 | 127.25 | 81.54 | 0.57 | 8.36 |
|  |  |  | SD | 25.91 | 73.63 | 97.74 | 24.92 | 2.43 | 0.11 | 1.23 |
|  |  |  | Range | 47.18-110.62 | 230.45-422.03 | 277.97-532.65 | 93.32-156.14 | 77.15-84.38 | 0.43-0.76 | 6.19-10.11 |
| MH | 19 |  | Mean | 54.45 | 200.53 | 254.98 | 90.79 | 78.66 | 0.59 | 10.24 |
|  |  |  | SD | 16.48 | 56.57 | 71.2 | 19.26 | 2.83 | 0.09 | 1.54 |
|  |  |  | Range | 32.14-90.83 | 123.44-351.01 | 155.57-441.84 | 64.89-136.08 | 71.02-82.88 | 0.46-0.83 | 8.45-14.83 |
|  |  |  |  |  |  |  |  |  |  |  |
| TD6 | 3 | M_3_ | Mean | 23.80 | 122.05 | 145.85 | 49.67 | 82.38 | 0.46 | 10.48 |
|  |  |  | SD | 16.18 | 93.05 | 109.22 | 31.70 | 2.71 | 0.05 | 2.72 |
|  |  |  | Range | 6.91-39.16 | 93.05-122.05 | 33.39-251.52 | 17.21-80.55 | 79.31-84.43 | 0.40-0.49 | 8.15-13.47 |
| HER | 1 |  |  | 29.41 | 145.13 | 174.54 | 64.21 | 83.15 | 0.46 | 8.72 |
| AT-30 |  |  |  | 35.34 | 136.76 | 172.10 | 62.92 | 79.47 | 0.56 | 10.90 |
| AT-811 |  |  |  | 53.27 | 194.36 | 247.63 | 90.32 | 78.49 | 0.59 | 10.18 |
| AT-143 |  |  |  | 46.77 | 159.00 | 205.77 | 72.33 | 77.27 | 0.65 | 11.94 |
| AT-1468 |  |  |  | 50.54 | 157.42 | 207.96 | 75.29 | 75.70 | 0.67 | 12.43 |
| AT-599 |  |  |  | 50.84 | 136.58 | 187.42 | 71.66 | 72.87 | 0.71 | 13.78 |
| AT-942 |  |  |  | 64.00 | 190.62 | 254.62 | 89.77 | 74.86 | 0.71 | 12.39 |
| AT-1959 |  |  |  | 42.45 | 156.58 | 199.03 | 50.21 | 78.67 | 0.85 | 15.69 |
| AT-2438b |  |  |  | 37.44 | 122.91 | 160.35 | 61.79 | 76.65 | 0.61 | 12.19 |
| AT-2273 |  |  |  | 38.98 | 136.47 | 175.45 | 64.02 | 77.78 | 0.61 | 11.83 |
| AT-2777 |  |  |  | 64.00 | 179.87 | 243.87 | 82.48 | 73.76 | 0.78 | 13.75 |
| AT-3182 |  |  |  | 56.30 | 170.83 | 227.13 | 76.29 | 75.21 | 0.74 | 13.30 |
| AT-3943 |  |  |  | 46.81 | 135.04 | 181.85 | 70.35 | 74.26 | 0.67 | 12.97 |
| **SH** | **12** |  | **Mean** | **48.90** | **156.37** | **205.27** | **72.29** | **76.25** | **0.68** | **12.61** |
|  |  |  | **SD** | **9.54** | **23.61** | **31.79** | **11.75** | **2.12** | **0.08** | **1.44** |
|  |  |  | **Range** | **35.34-64.00** | **122.91-194.36** | **160.35-254.62** | **50.21-90.32** | **72.87-79.47** | **0.56-0.85** | **10.18-15.69** |
| NAH_Tig | 1 |  |  | 79.89 | 302.65 | 382.54 | 124.33 | 79.12 | 0.64 | 9.57 |
| MPEH_M-LN | 1 |  |  | 68.05 | 207.24 | 275.29 | 88.75 | 75.28 | 0.77 | 12.96 |
| NEA | 6 |  | Mean | 66.21 | 241.39 | 307.60 | 111.12 | 78.44 | 0.59 | 9.59 |
|  |  |  | SD | 14.46 | 44.95 | 54.29 | 15.01 | 3.42 | 0.09 | 1.61 |
|  |  |  | Range | 48.02-83.44 | 195.19-314.03 | 259.42-394.36 | 97.61-138.28 | 73.59-81.49 | 0.49-0.72 | 8.26-11.65 |
| MH | 13 |  | Mean | 47.92 | 176.58 | 224.50 | 84.04 | 78.62 | 0.57 | 10.24 |
|  |  |  | SD | 16.65 | 60.43 | 75.60 | 21.75 | 3.15 | 0.10 | 2.01 |
|  |  |  | Range | 22.25-79.90 | 114.33-314.86 | 152.29-394.75 | 56.12-131.75 | 73.75-85.39 | 0.32-0.71 | 6.28-13.98 |

Upper molars: TD6: *H. antecessor* from Gran Dolina [41]. NAH: North African *Homo* (Tf: Tighenif [46]). AT & SH: Atapuerca-Sima de los Huesos (original data). EMPH: European Middle Pleistocene *Homo* (Vg: Visogliano [24]). NEA: Neanderthals [41]. LV: Lagar Velho (Original data from Nespos). MH: modern humans [41 and original data].

Lower molars: TD6: *H. antecessor* from Gran Dolina [41]. NAH: North African *Homo* (Tf:Tighenif [46]). AT & SH: Atapuerca-Sima de los Huesos (original data). HER: *H. erectus* (Sangiran, [45]). EMPH: European Middle Pleistocene *Homo* (FR: Fontana Ranuccio [24]). NEA: Neanderthals [41]. LV: Lagar Velho (Original data from Nespos). MH: modern humans [41 and original data].
