# Supplementary material for: Crown tissue proportions and enamel thickness distribution in the Middle Pleistocene hominin molars from Sima de los Huesos (SH) population (Atapuerca, Spain)
Source: PLoS One. 2020 Jun 8;15(6):e0233281. doi: 10.1371/journal.pone.0233281 (PMC7279586; doi:10.1371/journal.pone.0233281)
Supplement: S1 Data — (DOCX) [file pone.0233281.s009.docx]

Repository information

| Group/Species | Tooth | Specimen | Institution |
| --- | --- | --- | --- |
| Sima de los Huesos | M^1^ | AT-20 | Centro Mixto (UCM-ISCIII) de Evolución y Comportamiento Humanos, Madrid, Spain |
| Sima de los Huesos | M^1^ | AT-26 |  |
| Sima de los Huesos | M^1^ | AT-16 |  |
| Sima de los Huesos | M^1^ | AT-196 |  |
| Sima de los Huesos | M^1^ | AT-812 |  |
| Sima de los Huesos | M^1^ | AT-944 |  |
| Sima de los Huesos | M^1^ | AT-959 |  |
| Sima de los Huesos | M^1^ | AT-2071 |  |
| Sima de los Huesos | M^1^ | AT-3178 |  |
| Sima de los Huesos | M^1^ | AT-3177 |  |
| Sima de los Huesos | M^1^ | AT-5804 |  |
| *Homo sapiens* | M^1^ | UCM31_36 | Instituto de Medicina Legal, Universidad Complutense de Madrid, Spain |
| *Homo sapiens* | M^1^ | UCM38_26 |  |
| *Homo sapiens* | M^1^ | UCM57_16 |  |
| *Homo sapiens* | M^1^ | UCM76_16 |  |
| *Homo sapiens* | M^1^ | UCM38_16 |  |
| *Homo sapiens* | M^1^ | UCM57_26 |  |
| *Homo sapiens* | M^1^ | UCM49_26 |  |
| *Homo sapiens* | M^1^ | UCM44_26 |  |
| Sima de los Huesos | M^2^ | AT-12 | Centro Mixto (UCM-ISCIII) de Evolución y Comportamiento Humanos, Madrid, Spain |
| Sima de los Huesos | M^2^ | AT-824 |  |
| Sima de los Huesos | M^2^ | AT-817 |  |
| Sima de los Huesos | M^2^ | AT-4326 |  |
| Sima de los Huesos | M^2^ | AT-270 |  |
| Sima de los Huesos | M^2^ | AT-15 |  |
| Sima de los Huesos | M^2^ | AT-170 |  |
| Sima de los Huesos | M^2^ | AT-960 |  |
| Sima de los Huesos | M^2^ | AT-822 |  |
| Sima de los Huesos | M^2^ | AT-2175 |  |
| Sima de los Huesos | M^2^ | AT-815 |  |
| Sima de los Huesos | M^2^ | AT-588 |  |
| Sima de los Huesos | M^2^ | AT-4336 |  |
| Sima de los Huesos | M^2^ | AT-6215 |  |
| *Homo sapiens* | M^2^ | UCM14_17 | Instituto de Medicina Legal, Universidad Complutense de Madrid, Spain |
| *Homo sapiens* | M^2^ | UCM38_17 |  |
| *Homo sapiens* | M^2^ | UCM39_17 |  |
| *Homo sapiens* | M^2^ | UCM44_27 |  |
| *Homo sapiens* | M^2^ | UCM63_17 |  |
| *Homo sapiens* | M^2^ | UCM65_17 |  |
| *Homo sapiens* | M^2^ | UCM9_27 |  |
| Sima de los Huesos | M^3^ | AT-10 | Centro Mixto (UCM-ISCIII) de Evolución y Comportamiento Humanos, Madrid, Spain |
| Sima de los Huesos | M^3^ | AT-194 |  |
| Sima de los Huesos | M^3^ | AT-601 |  |
| Sima de los Huesos | M^3^ | AT-805 |  |
| Sima de los Huesos | M^3^ | AT-826 |  |
| Sima de los Huesos | M^3^ | AT-3181a |  |
| Sima de los Huesos | M^3^ | AT-1471 |  |
| Sima de los Huesos | M^3^ | AT-2393 |  |
| Sima de los Huesos | M^3^ | AT-3183 |  |
| Sima de los Huesos | M^3^ | AT-5082 |  |
| Sima de los Huesos | M^3^ | AT-5292 |  |
| Sima de los Huesos | M^3^ | AT-274 |  |
| Sima de los Huesos | M^3^ | AT-602 |  |
| Sima de los Huesos | M^3^ | AT-6215 |  |
| *Homo sapiens* | M^3^ | UCM36_18 | Instituto de Medicina Legal, Universidad Complutense de Madrid, Spain |
| *Homo sapiens* | M^3^ | UCM44_28 |  |
| *Homo sapiens* | M^3^ | UCM66_18 |  |
| *Homo sapiens* | M^3^ | UCM38_28 |  |
| *Homo sapiens* | M^3^ | UCM16_18 |  |
| *Homo sapiens* | M^3^ | UCM68_28 |  |
| *Homo sapiens* | M^3^ | UCM20_28 |  |
| Sima de los Huesos | M_1_ | AT-2 | Centro Mixto (UCM-ISCIII) de Evolución y Comportamiento Humanos, Madrid, Spain |
| Sima de los Huesos | M_1_ | AT-3933 |  |
| Sima de los Huesos | M_1_ | AT-101 |  |
| Sima de los Huesos | M_1_ | AT-141 |  |
| Sima de los Huesos | M_1_ | AT-272 |  |
| Sima de los Huesos | M_1_ | AT-829 |  |
| Sima de los Huesos | M_1_ | AT-1759 |  |
| Sima de los Huesos | M_1_ | AT-2276 |  |
| Sima de los Huesos | M_1_ | AT-2438 |  |
| Sima de los Huesos | M_1_ | AT-4318 |  |
| Sima de los Huesos | M_1_ | AT-21 |  |
| Sima de los Huesos | M_1_ | AT-576 |  |
| Sima de los Huesos | M_1_ | AT-561 |  |
| *Homo sapiens* | M_1_ | SP973_36 | PACEA, Université de Bordeaux, France |
| *Homo sapiens* | M_1_ | SP965_46 |  |
| *Homo sapiens* | M_1_ | UCM44_46 | Instituto de Medicina Legal, Universidad Complutense de Madrid, Spain |
| *Homo sapiens* | M_1_ | UCM51_46 |  |
| *Homo sapiens* | M_1_ | UCM53_46 |  |
| *Homo sapiens* | M_1_ | UCM79_36 |  |
| *Homo sapiens* | M_1_ | UCM38_46 |  |
| *Homo sapiens* | M_1_ | UCM16_36 |  |
| Sima de los Huesos | M_2_ | AT-3179 | Centro Mixto (UCM-ISCIII) de Evolución y Comportamiento Humanos, Madrid, Spain |
| Sima de los Huesos | M_2_ | AT-169 |  |
| Sima de los Huesos | M_2_ | AT-271 |  |
| Sima de los Huesos | M_2_ | AT-284 |  |
| Sima de los Huesos | M_2_ | AT-1761 |  |
| Sima de los Huesos | M_2_ | AT-941 |  |
| Sima de los Huesos | M_2_ | AT-946 |  |
| Sima de los Huesos | M_2_ | AT-2270 |  |
| Sima de los Huesos | M_2_ | AT-2396 |  |
| Sima de los Huesos | M_2_ | AT-3176 |  |
| Sima de los Huesos | M_2_ | AT-3176 |  |
| Sima de los Huesos | M_2_ | AT-6579 |  |
| *Homo sapiens* | M_2_ | SP50_37 | PACEA, Université de Bordeaux, France |
| *Homo sapiens* | M_2_ | UCM44_37 | Instituto de Medicina Legal, Universidad Complutense de Madrid, Spain |
| *Homo sapiens* | M_2_ | UCM9_37 |  |
| *Homo sapiens* | M_2_ | UCM69_37 |  |
| *Homo sapiens* | M_2_ | AC33_37_H | Centro Nacional de Investigación sobre la Evolución Humana (CENIEH), Burgos, Spain |
| *Homo sapiens* | M_2_ | AG1_47 |  |
| *Homo sapiens* | M_2_ | AC33_37 |  |
| *Homo sapiens* | M_2_ | CR20080000017 |  |
| *Homo sapiens* | M_2_ | R5804_37 |  |
| Sima de los Huesos | M_3_ | AT-30 | Centro Mixto (UCM-ISCIII) de Evolución y Comportamiento Humanos, Madrid, Spain |
| Sima de los Huesos | M_3_ | AT-811 |  |
| Sima de los Huesos | M_3_ | AT-143 |  |
| Sima de los Huesos | M_3_ | AT-1468 |  |
| Sima de los Huesos | M_3_ | AT-599 |  |
| Sima de los Huesos | M_3_ | AT-942 |  |
| Sima de los Huesos | M_3_ | AT-1959 |  |
| Sima de los Huesos | M_3_ | AT-2438b |  |
| Sima de los Huesos | M_3_ | AT-2273 |  |
| Sima de los Huesos | M_3_ | AT-2777 |  |
| Sima de los Huesos | M_3_ | AT-3182 |  |
| Sima de los Huesos | M_3_ | AT-3943 |  |
| *Homo sapiens* | M_3_ | CR20080000092_38 | Centro Nacional de Investigación sobre la Evolución Humana (CENIEH), Burgos, Spain |
| *Homo sapiens* | M_3_ | CR20080000123_38 |  |
| *Homo sapiens* | M_3_ | UCM18_38 | Instituto de Medicina Legal, Universidad Complutense de Madrid, Spain |
| *Homo sapiens* | M_3_ | UCM31_38 |  |
| *Homo sapiens* | M_3_ | UCM37_48 |  |
| *Homo sapiens* | M_3_ | UCM65_38 |  |
| *Homo sapiens* | M_3_ | UCM4_48 |  |
